# A Field‐Deployable eDNA Metabarcoding Workflow Including De Novo Reference Assembly for Characterising Understudied Biodiversity Hotspots

**DOI:** 10.1111/1755-0998.70122

**Published:** 2026-03-31

**Authors:** Jesse Erens, Christopher Heine, Stefan Lötters, Henrik Krehenwinkel, Andrew J. Crawford, Luis Alberto Rueda‐Solano, Amadeus Plewnia

**Affiliations:** ^1^ Biogeography Department Trier University Trier Germany; ^2^ Habitats Conservation Hostert Luxembourg; ^3^ Marine Evolution and Ecology Group, Naturalis Biodiversity Center Leiden the Netherlands; ^4^ Department of Biological Sciences Universidad de los Andes Bogotá Colombia; ^5^ Facultad de Ciencias Básicas, Universidad del Magdalena Santa Marta Colombia

**Keywords:** amphibians, environmental DNA, metagenomics, Oxford nanopore sequencing, recombinase polymerase amplification, third‐generation sequencing

## Abstract

Field‐deployable DNA metabarcoding offers a transformative approach to biodiversity research and monitoring, yet its application remains limited due to technical constraints and a lack of reference data in poorly studied ecosystems. Combining isothermal Recombinase Polymerase Amplification (RPA) and Oxford Nanopore sequencing, we introduce a two‐step approach that uses non‐invasive species barcoding to directly generate reference sequences for use in environmental DNA (eDNA) metabarcoding, and enables real‐time, PCR‐free and cost‐effective molecular assessment of ecological communities in the field. Using an endemic and understudied tropical amphibian assemblage as a model, we demonstrate the functionality of this novel workflow. De novo generation of a reference sequence library from amphibian skin swab samples significantly improved the accuracy and taxonomic resolution of sequence assignments from eDNA samples, particularly on the species level, in turn allowing a characterisation of fine‐scale patterns in community composition. Beyond generating new RPA‐compatible amphibian metabarcoding primers, our results show that combining field‐based eDNA metabarcoding with the offline assembly of a local reference database can directly bridge existing data gaps in molecular biodiversity monitoring, providing a scalable solution to accelerate biodiversity assessments in data‐deficient ecosystems. This workflow paves the way for broader deployment of molecular tools in global biodiversity hotspots—particularly in remote and resource‐limited tropical regions—to directly contribute critical baseline data, and support conservation efforts in regions where they are most urgently needed.

## 1 Introduction

In the face of rapid and ongoing biodiversity decline, improved access to field‐deployable, cost‐effective and non‐invasive molecular species detection tools is of primary importance (Kühl et al. 2020; Theissinger et al. 2023; Plewnia, Hildwein, et al. 2026). Since global resources for biodiversity conservation remain limited, it becomes essential to be able to prioritise species and communities to achieve timely and effective conservation action (Wilson et al. 2006). Among the key limitations that currently hamper our efforts to preserve wildlife populations, however, are a lack of knowledge on how species are exactly distributed and how many taxa occur within a given area (Bini et al. 2006; Brito 2010). These shortfalls are most severe in tropical regions, the very areas that harbour the highest levels of biodiversity but often lack sufficient resources for comprehensive research and conservation (Collen et al. 2008). Hence, there is an acute need to make molecular biodiversity monitoring methods more accessible and cost‐efficient to enable their wider application in understudied and hyperdiverse tropical ecosystems.

Community metabarcoding is a rapidly advancing toolkit employed in the standardised assessment of biodiversity. Its application enables comprehensive assessments of biodiversity change and community composition across scales ranging from microbes to entire ecosystems (Compson et al. 2020; Creer et al. 2016; Gillespie et al. 2023). The development of portable and lower‐cost laboratory equipment and high‐throughput sequencing technologies is currently democratising molecular biodiversity monitoring (Hatfield et al. 2023; Krehenwinkel et al. 2019; LaBarre et al. 2011; Ríos et al. 2012; Plewnia et al. 2025), making the metabarcoding toolkit increasingly accessible to conservationists, practitioners and researchers in remote, resource‐limited settings. Presently, multi‐step DNA extraction kits can, for instance, be replaced with a fast and simplified cellulose‐based extraction protocol that yields lowered but sufficient DNA for metabarcoding applications (Zou et al. 2018; Plewnia et al. 2025). By further using Recombinase Polymerase Amplification (RPA) as a rapid (< 30 min) and isothermal (~37°C) alternative to Polymerase Chain Reaction (PCR), and using a portable Oxford Nanopore Technology sequencing device (hereafter: ‘Nanopore’) instead of laboratory‐based DNA sequencing machines, large equipment and traditional laboratory infrastructures can be bypassed entirely (Plewnia et al. 2025). However, field‐deployable metabarcoding is still in its infancy, and several significant limitations hinder its broader application, including for environmental DNA (eDNA) studies.

First, constraints remain due to the specific requirements for RPA primer design (Li et al. 2019; Lobato and O'Sullivan 2018). RPA performs best with long primers (~30 bases), which are prone to artefact formation (Lobato and O'Sullivan 2018), which challenges the design of such long primers for higher taxa that commonly share only short conserved regions. For eDNA metabarcoding, primer design is further limited to the few, mostly mitochondrial markers for which broad sequence libraries are available to serve as a reference for taxonomic assignment. Nevertheless, RPA has shown greater tolerance to sequence mismatches at primer binding sites (Daher et al. 2015), increasing its potential applicability across closely related species. Thus, the emergence of RPA introduces both new challenges and opportunities, and an experimental approach is still required to assess the compatibility of existing PCR primers. As such, general guidelines and analytical tools for adapting PCR primers for RPA‐based metabarcoding are still largely lacking.

Second, the incompleteness and inaccuracy of DNA reference databases, particularly for the tropical sampling gap, presently limits molecular taxonomic identification of Operational Taxonomic Units (OTUs) from environmental samples (Hughes et al. 2021; Jetz et al. 2012). The development of portable sequencing devices, with the Nanopore MinION being the most widely used platform, now allows high‐throughput DNA barcoding of hundreds of specimens with minimal equipment in the field (Krehenwinkel et al. 2019; Pomerantz et al. 2022). Complementing field‐deployable eDNA metabarcoding with simultaneous reference database creation from targeted species‐specific barcoding could therefore substantially increase taxonomic accuracy and accelerate the real‐time characterisation of ecological communities in the field.

In this paper, we address these limitations and expand the RPA‐based metabarcoding workflow. We do this by (1) nesting a novel environmental DNA (eDNA) metabarcoding primer set within a long‐read specimen barcoding marker. This enables fully isothermal, field‐deployable community metabarcoding from environmental samples with species identification being guided by (2) a reference database that is de novo assembled using non‐invasive DNA sampling methods and the same sequencing workflow as used for environmental samples. We demonstrate the real‐world application of this novel approach through the molecular characterisation of an understudied tropical montane amphibian community of high conservation relevance. By further expanding open‐source protocols for RPA primer development for metabarcoding and local, offline reference database assemblies, we facilitate the application of field‐based metabarcoding to advance global efforts in biodiversity assessment and conservation.

## 2 Materials and Methods

### 2.1 RPA Primer Design

Our eDNA assay targets the 16S ribosomal RNA gene, one of the most widely used genetic markers for barcoding amphibians (Kocher et al. 1989; Palumbi et al. 2002; Salducci et al. 2005; Vences et al. 2005), making it highly suitable for eDNA metabarcoding (Sakata et al. 2022). We started with various previously utilised PCR primers designed for different amplicons within 16S and further modified primers by manually adding bases to one or both ends to increase their potential for RPA compatibility. As the foundation for primer design, we used the recent 16S alignment of Portik et al. (2023), which includes sequence data for 4890 anuran species. As we visually identified misaligned regions in the dataset, we re‐aligned all 16S sequences using MAFFT (Katoh and Standley 2013) and removed insertions and divergent regions with CIAlign (Tumescheit et al. 2022) as well as regions with > 50% gaps in all sequences using trimAL (Capella‐Gutiérrez et al. 2009). We then aligned existing primers (Palumbi et al. 2002; Sakata et al. 2022) and visually screened for additional conserved regions and conserved positions bordering the existing primer binding sites. Based on the identified conserved positions, we then elongated existing primers to a total of ~25–30 bases while minimising the number of degenerate bases. In addition to the ‘universal’ 16S primer 16Sbr (Palumbi et al. 2002), we designed a total of 24 new candidate RPA primers spread across six different binding sites along the 16S gene for further testing with RPA (Table S1), with various target amplicon lengths (Figure S1). We assessed the RPA functionality of 77 primer combinations using amphibian DNA isolates from tissue samples (given material constraints, we could not test all primer combinations). This yielded 29 pairs that showed visually consistent amplification based on gel electrophoresis, which were selected for further testing amplification success from eDNA samples (Figure S2). The five candidate primer pairs with the strongest visual amplification of eDNA samples based on gel electrophoresis were taken for further Nanopore indexing and a test MinION sequencing run (Figure S2). For dual barcoding of each sample, we added 20‐base indexes adopted from Gajski et al. (2024) to the 5′ end of each selected primer. Selected primers (including indexes) were checked in silico for potential dimers and hairpin structures at relevant thermal conditions using the Multiple Primer Analyser (Thermo Fisher) and OligoAnalyzer (IDT).

We finally selected the primer pair MiAmphiL_28F (5′‐CCTCGCCTGTTTACCAAAAAC**AYCGCCT**‐3′) ‐ MiAmphiL_extR (5′‐**AAG**CTCCAT**R**GGGTCTTCTCGT**CTWRT**‐3′) (Bases indicated in bold were added or modified from the primers of Sakata et al. 2022) to generate short‐read amplicons (~250 bp) from environmental samples. The primer pair MiAmphiL_28F—16Sbr (5′‐CCGGTCTGAACTCAGATCACGT‐3′), combining the same forward primer with the ‘universal’ 16Sbr primer, was selected for obtaining long‐read barcodes from skin swab samples collected from amphibian specimens. See Protocol S1 in the Supporting Information for additional recommendations on the design of RPA‐compatible metabarcoding primers.

### 2.2 RPA Assay Validation

We used the TwistAmp basic kit (TwistDX, Abbott) for RPA following the manufacturer's instructions with deviations and two‐step indexing for high‐throughput sequencing as described in Plewnia et al. (2025). In brief, the amphibian target fragment was amplified with a completely homologous version of the respective primers in a first RPA. In a subsequent RPA, these initial amplicons were amplified with a version of the same primer pair now carrying non‐homologous 20‐bp indexes as RPA's more localised DNA strand‐displacement does not allow for index introduction in a single step. In addition, for environmental samples (in this case: eDNA water samples), we increased the template volume to 2.64 μL and did not add nuclease‐free water to keep the reaction volume of 10 μL constant for the first RPA, while for the indexing RPA we used 1 μL template only. A negative control was included to help account for background contamination (see sequence processing below). RPA products were screened with gel electrophoresis for successful amplification.

### 2.3 Library Preparation and Sequencing

Indexed RPA products were pooled at equal volumes and cleaned using magnetic beads (NucleoMag, Macherey‐Nagel). We prepared libraries using the SQK‐LSK114 kit following the manufacturer's instructions for Flongle libraries. However, we changed the input volume, using 200 ng of DNA from the cleaned and combined pools as we previously experienced that the recommended 50–100 fmol led to high proportions of inactive pores when sequencing short amplicons. We further deviated cycling conditions for end‐prep, incubating for 30 min at 20°C and 30 min at 65°C. Sequencing was conducted for 20–30 h depending on pore activity on Nanopore FLO‐FLG114 Flongle flow cells using R10.4 chemistry and following the manufacturer's instructions. For a detailed field‐deployable protocol, please refer to Protocol S2 in the Supporting Information.

### 2.4 Sequence Processing and Field‐Based Reference Assembly

We employed Dorado 0.9.0 for GPU‐based basecalling of MinION reads using the super‐accurate (sup) model. Demultiplexing and trimming of sequence reads was done using minibar (Krehenwinkel et al. 2019) with default edit distances, which we observed to be conservative regarding sequence misassignment but not leading to excessive sequence loss (settings may need adjustment given different index lengths and sequence error rates). We then filtered sequences for average Phred scores > 12 and read length of 172–212 bp for the eDNA marker and 520–580 bp for the barcoding marker using SeqKit (Shen et al. 2024). As we expected the community structure to be complex, including closely related species and unequal abundances for the eDNA marker, we refrained from OTU clustering approaches. Instead, we simply dereplicated identical sequences using Vsearch (Rognes et al. 2016) and mapped all unique sequences to a reference database following the direct BLASTn approach (Plewnia et al. 2025). We chose the MIDORI2 reference database (MIDORI_UNIQ_NUC_SP_GB263_lrRNA_BLAST, based on a GenBank download from 13 October 2024; Leray et al. 2022), which is a readily available reference database for the long ribosomal subunit that contains all unique but no duplicated sequences from GenBank. With its small size, this database allows offline taxonomic assignment on a local machine without connectivity or vast computational resources. For sequence assignment, we used BLASTn through BLAST+ (v 2.16.0). BLASTn was set to select and keep only the first (i.e., best) taxonomic hit to avoid arbitrary selection of taxa. As the MIDORI2 database already contains detailed taxonomic information, no subsequent ‘translation’ of accession numbers into species names was required. Taxonomic hits were filtered at both a 95% and 99% sequence identity threshold. Since no standardised threshold exists to delineate amphibian taxa and results may differ depending on the study system, these values were set experimentally to compare read assignments at a stringent and more permissive setting. Read counts ≤ 5 for any given taxa were regarded as noise and set to 0 in each sample, and taxa that were present in the negative control were removed from the final results to mitigate contamination. Note that while we here used one negative amplification control, a broader set of both negative field and amplification controls may be used to account for contamination more extensively.

For single‐specimen barcoding, we built consensus sequences from filtered reads using NGSpeciesID (Sahlin et al. 2021), with the ‐‐ont option optimised for Nanopore reads and the medaka polishing algorithm (see Protocol S3 for details). Because NGSpeciesID may produce erroneous consensus sequences when target sequence abundance is low (pers. obs.), we visually examined all consensus sequences by aligning sequences using MAFFT with automated reverse complementing and subsequent polishing with CIAlign as described above. Consensus sequences were then manually trimmed in the alignment to remove divergent sequence ends that are sometimes erroneously introduced by NGSpeciesID. We subsequently inferred a maximum‐likelihood phylogenetic tree using IQ‐TREE 2 with 100,000 ultrafast bootstrap replicates and 100,000 replicates of the SH‐like approximate likelihood ratio test (Minh et al. 2020), rooted with the caudate amphibian sequences generated (Table S2), to further validate the integrity of the curated consensus sequences (Figure S3). Nanopore MinION sequencing reads are nevertheless noted to involve higher raw sequencing error rates than other platforms (Delahaye and Nicolas 2021; Pomerantz et al. 2022; Ohta et al. 2023). We therefore also included previously available Sanger sequencing data in this phylogenetic comparison to assess potential inconsistencies related to different sequencing platforms, and whether Nanopore (R10.4) error rates may affect species‐level identification (Figure S3; Table S3).

The newly obtained 16S barcoding sequences were stored as FASTA files with species name, unique identifier and sequence data stored in the header for incorporation in the reference database. Sequences were then concatenated into the FASTA‐formatted version of the same MIDORI2 database and indexed into a searchable database using BLAST+. Subsequently, eDNA reads were blasted against the manually supplemented database as described above. Provided a moderately powerful laptop computer (16–32 GB RAM, 8–12 core processor) and a dataset comparable to our present study, basecalling will take ~2–3 days on a CPU, and ~5–10 h when run on a GPU. A further ~2–4 h will be needed for the various read filtering and processing steps. Using a custom, locally stored mitochondrial reference database (in our case: MIDORI2 complemented with local reference barcodes) will strongly reduce the computational time needed for blasting the eDNA reads compared to using the complete GenBank nt database, and generally requires less than 30 min.

GenBank accession numbers for single‐specimen 16S barcoding data generated in this paper are available in Table S2. Basecalled and demultiplexed raw MinION reads from both environmental samples and specimens are available in the supporting data (https://doi.org/10.6084/m9.figshare.28417451). Associated scripts for building the integrated reference database and sequence processing are provided in Protocol S3 and Protocol S4 in the Supporting Information. Refer to Protocol S5 for an overview of material requirements and considerations for carrying out the workflow under field conditions.

### 2.5 Case Study

To validate the performance of our eDNA assay, we collected water from 13 streams in the Sierra Nevada de Santa Marta of Colombia (see Figure S4; Table S4). This isolated mountain range is home to a diverse community of locally endemic amphibian species (Ruthven 1922; Pérez‐González et al. 2015; Rueda‐Solano et al. 2016). Due to its remoteness and limited accessibility, the entire region remains under‐researched and sequences for taxonomic reference are only available for a subset of species (Arroyo et al. 2022; Lötters et al. 2025), which has so far impeded metagenetic biodiversity assessments. eDNA (water) samples were taken on site using a portable, self‐manufacturable pump system (see Plewnia et al. 2025). In each stream, water was filtered through 0.45 μm nitrocellulose filters (47 mm disks, Sartorius) in a 250 mL funnel (Nalgene, Thermo Scientific) until the filter clogged. Filters were handled using fire‐sterilised tweezers and filtering was done using sterile nitrile gloves to minimise contamination. Filters were stored in DNA/RNA Shield (Zymo Research) after sampling. In addition to the eDNA samples, we collected non‐invasive skin swabs from all encountered amphibian species at the same sampling sites, which were used to create a de novo reference database as described above. Amphibians were surveyed opportunistically at each sampling locality both by visual and acoustic observation for a total of around 1 h of effective searching time (as part of a larger inventory; Plewnia, Pasmans, et al. 2026). Skin swabs were taken by stroking a cotton‐tipped swab ~20 times across the ventral surface of each individual, after which the swab tip was stored in DNA/RNA Shield. Encountered individuals were photographed, and original species descriptions were consulted when needed to verify identifications. Identifications were finally cross‐validated with barcoding results as described above (Figure S3).

Samples were extracted using the DNeasy Blood and Tissue Kit (Qiagen) following the protocol of Plewnia et al. (2025) for water filters and Böning et al. (2024) for amphibian swab samples. As the swab samples were also used for a parallel study on the same study system, we did not employ cellulose‐based DNA ‘fast‐extraction techniques’ directly in the field. However, since we have shown previously that these techniques can yield sufficient RPA‐ready template under field conditions for both amphibian skin swabs (Hoenig et al. 2023, 2024) and environmental samples (Plewnia, Krehenwinkel, and Heine 2025), our workflow remains entirely field‐deployable (see Protocols S2–S5 in the Supporting Information). We classified species and genera as ‘incorrectly assigned’ from eDNA when they were neither visually observed and identified in our sampling sites during the present study, nor referenced in any other studies (Ruthven 1922; Pérez‐González et al. 2015; Rueda‐Solano et al. 2016; Plewnia, Pasmans, et al. 2026). Considering the near‐complete assignment of eDNA reads to observed species using our de novo reference assembly (see Section 3), this approach was deemed unlikely to falsely disregard undescribed amphibian diversity in our eDNA samples.

## 3 Results

### 3.1 An RPA and Nanopore‐Based Workflow for Field‐Deployable Parallel Species Barcoding and eDNA Metabarcoding

**FIGURE 1:**
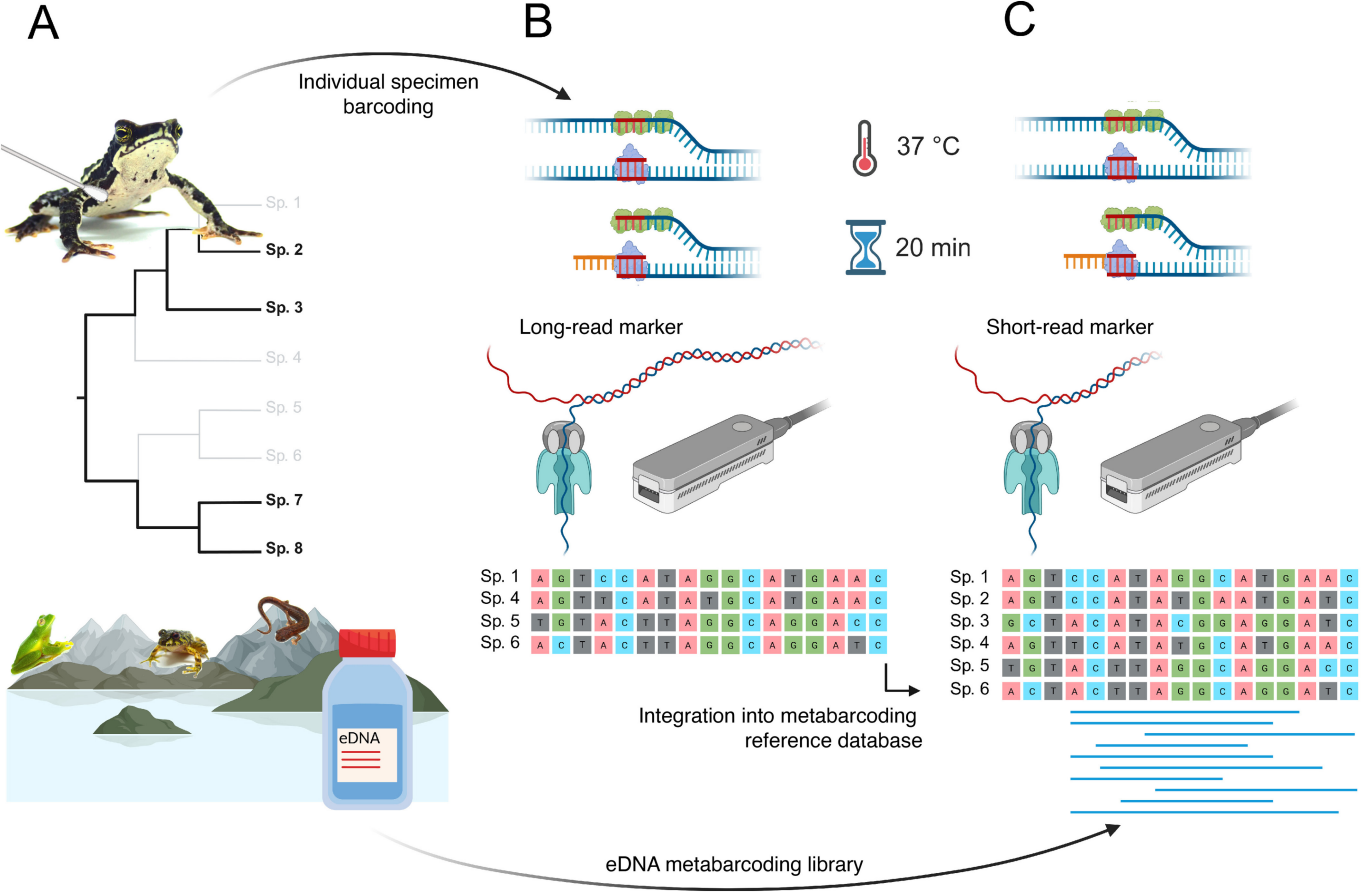
Overview of the novel RPA‐based metabarcoding workflow with de novo assembly of a reference database using a two‐step approach. (A) Environmental DNA (eDNA) sample collection is combined with non‐invasive DNA sampling of target species that are absent or under‐represented in existing reference datasets. Taxa theoretically lacking molecular reference data are displayed in grey in the phylogeny. (B) RPA‐based amplification and indexing is combined with Oxford Nanopore MinION sequencing to generate long‐read (~600 bp) consensus sequences for barcoding individual specimens. (C) eDNA samples are prepared in parallel for high‐throughput Oxford Nanopore MinION sequencing using a nested, short‐read marker (~250 bp). The resulting eDNA metabarcoding library is finally blasted to a reference database that is composed of a GenBank rRNA subset supplemented with the consensus sequences generated in (B). In the RPA illustration, the green protein represents the single‐stranded binding protein and the purple protein represents the recombinase.

A schematic summary of the integrative workflow is presented in Figure 1. The workflow combines isothermal library preparation from environmental samples with non‐invasive specimen barcoding for parallel on‐site sequencing using portable Nanopore platforms. Consensus sequences from specimen barcoding can directly be used to supplement or build a site‐specific reference database for offline and field‐deployable taxonomic assignment of the sequences generated from environmental samples. RPA was able to amplify long fragments (~600 bp) of 16S from amphibian skin swab samples, while short markers (~250 bp) were most suitable for amplifying anuran eDNA from environmental samples (Figure S2).

For single‐specimen samples, demultiplexed sequence reads comprised 128,679 total reads and a mean 1369 ± 2342 (150–20,880) per indexed sample, and 6748 total reads and a mean 72 ± 100 (8–542) per indexed sample after quality filtering (used in consensus sequence formation). For eDNA samples, demultiplexed sequence reads constituted 188,807 total reads and a mean 4720 ± 3277 (366–13,978) per indexed sample, and 137,132 total reads and a mean 3428 ± 3014 (126–11,844) per indexed sample after quality filtering (used in BLASTn). Complete field protocols and scripts used for sequence processing are provided in Protocols S2–S4 in the Supporting Information.

### 3.2 De Novo Reference Database Generation Improves Metabarcoding Accuracy

Based on non‐invasive DNA sampling of all amphibian morphospecies encountered at the eDNA sampling localities, we identified 17 taxa based on 72 sampled individuals (Table S2; Figure S3). For 12 of these taxa, at least one comparative 16S sequence was available prior to our study (Figure S3). The newly generated Nanopore consensus sequences were highly congruent with one another as well as with available Sanger sequences of the same species when compared to interspecific variation (Figure S3; Table S3), indicating that Nanopore sequencing error rates were unlikely to cause species misassignments.

**FIGURE 2:**
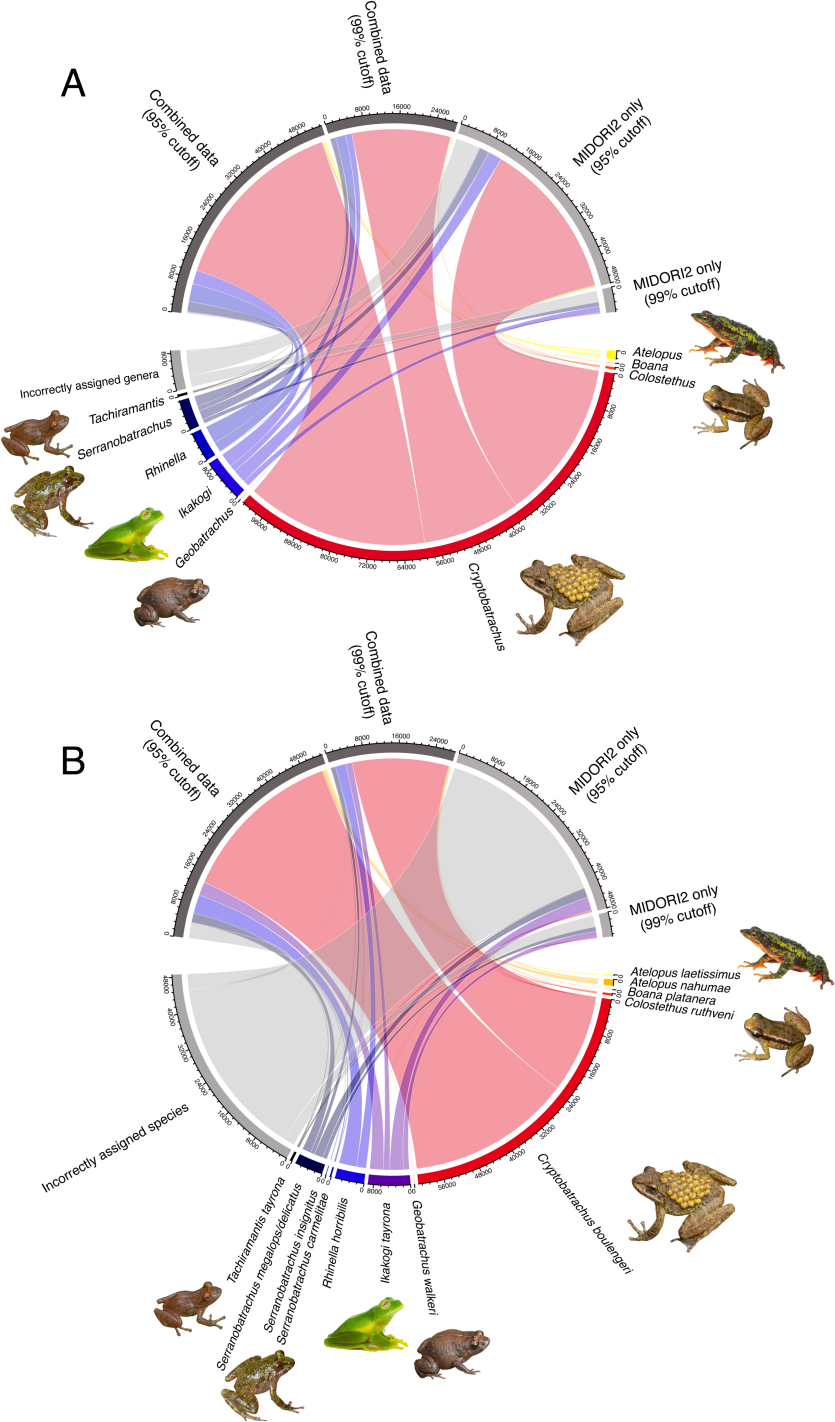
Summary of environmental DNA (eDNA) sequence reads, as obtained through isothermal library preparation and Nanopore sequencing and assigned to Operational Taxonomic Units (OTUs), based on 13 sampling sites in the Sierra Nevada de Santa Marta, northern Colombia. Two reference databases are compared, ‘MIDORI2’ and the ‘Combined data’ with new local specimen barcodes integrated into the MIDORI2 database. Comparisons use both a 95% and 99% sequence identity (BLASTn) cutoff for accepting the assigned taxonomy, with results summarised on (A) genus and (B) species level. Numbers correspond to the summed sequence reads that were assigned to amphibian taxa above each cutoff and given each database format, as well as the amphibian reads from each database that were correctly assigned to taxa (in colour) and those left incorrectly assigned (in grey) across all samples. Incorrectly assigned taxa are those that have not been recorded in the study area.

While we found that using prior available DNA reference data (MIDORI2; a mitochondrial rRNA GenBank subset) as a reference database for subsequent eDNA metabarcoding allowed a generalised taxonomic assignment at the genus level (Figure 2A), de novo assembly of a combined, site‐specific reference database (MIDORI2 integrated with the local single‐specimen barcoding data from 72 individuals) enabled a significantly improved taxonomic accuracy, most notably at the species level (Figure 2B).

At the 95% sequence similarity cutoff, amphibian reads constituted 61.8% (53,225 reads) of all reads above the threshold (total: 86,087) using the combined dataset and 59.4% (48,091 reads) of all reads above the threshold (total: 80,987) using the MIDORI2‐only dataset. The combined dataset yielded 99.5% correct genus‐level and 93.1% species‐level assignment of amphibian reads, compared to 88.3% and 12.2% for the MIDORI2‐only dataset, respectively. This corresponds to a 13% higher probability of correct read assignment at the genus level (RR = 1.13, 95% CI: 1.12–1.31, *p* < 0.001) but a more than seven times greater likelihood of correct species‐level assignment (RR = 7.63, 95% CI: 7.45–7.82, *p* < 0.001) when using the combined dataset. At this cutoff, the MIDORI2‐only dataset identified 6 visually confirmed species, whereas the combined dataset identified 12 species.

At the more stringent 99% sequence similarity cutoff, amphibian reads constituted 68.9% (28,302 reads) of all reads above the threshold (total: 41,074) using the combined dataset and 29.4% (5329 reads) of all reads above the threshold (total: 18,103) using the MIDORI2‐only dataset. The combined dataset showed a near‐complete correct assignment of amphibian reads (99.8%), with all correct genus‐level reads also resolving to the species level. The MIDORI2‐only dataset showed a substantially lower taxonomic accuracy (genus: 50.9%; species: 50.7%), meaning the probability of correct genus‐ and species‐level assignment was around twice as high when using the combined dataset (genus: RR = 1.96, 95% CI: 1.91–2.01, *p* < 0.001; species: RR = 1.97, 95% CI: 1.92–2.02; *p* < 0.001). At this cutoff, the MIDORI2‐only dataset identified 5 visually confirmed species, while the combined dataset identified 10 species.

Non‐amphibian OTUs of other locally known taxa largely corresponded to other vertebrates, with reads most commonly assigned to common opossum ( *Didelphis marsupialis* ), catfish (*Trichomycterus* spp., *Cordylancistrus tayrona*), the Colombian red howler monkey ( *Alouatta seniculus* ) and cattle ( *Bos taurus* ; in the lower regions). For an overview of total read counts and taxon assignments across the different field sites and reference database formats, please refer to the supporting data (https://doi.org/10.6084/m9.figshare.28417451).

### 3.3 De Novo Reference Database Construction Allows Real‐Time Characterisation of Community Structure

We employed the novel assay across 13 eDNA sampling localities situated at various elevations in the Sierra Nevada de Santa Marta mountain range in northern Colombia. By integrating local single‐specimen barcoding data into the reference database prior to eDNA analyses, a total of 12 visually confirmed amphibian species could be recovered in these environmental samples (using a 95% sequence similarity cutoff; Figure 2B). The inclusion of field and RPA replicates notably increased species detection probabilities across localities, with a mean 1.74 amphibian species detected per indexed sample, but a mean 2.92 per field site (~68% increase). This increase was 56% attributable to field sample replication and 44% to RPA replication (but based on varying replicates per field site; see Table S4).

**FIGURE 3:**
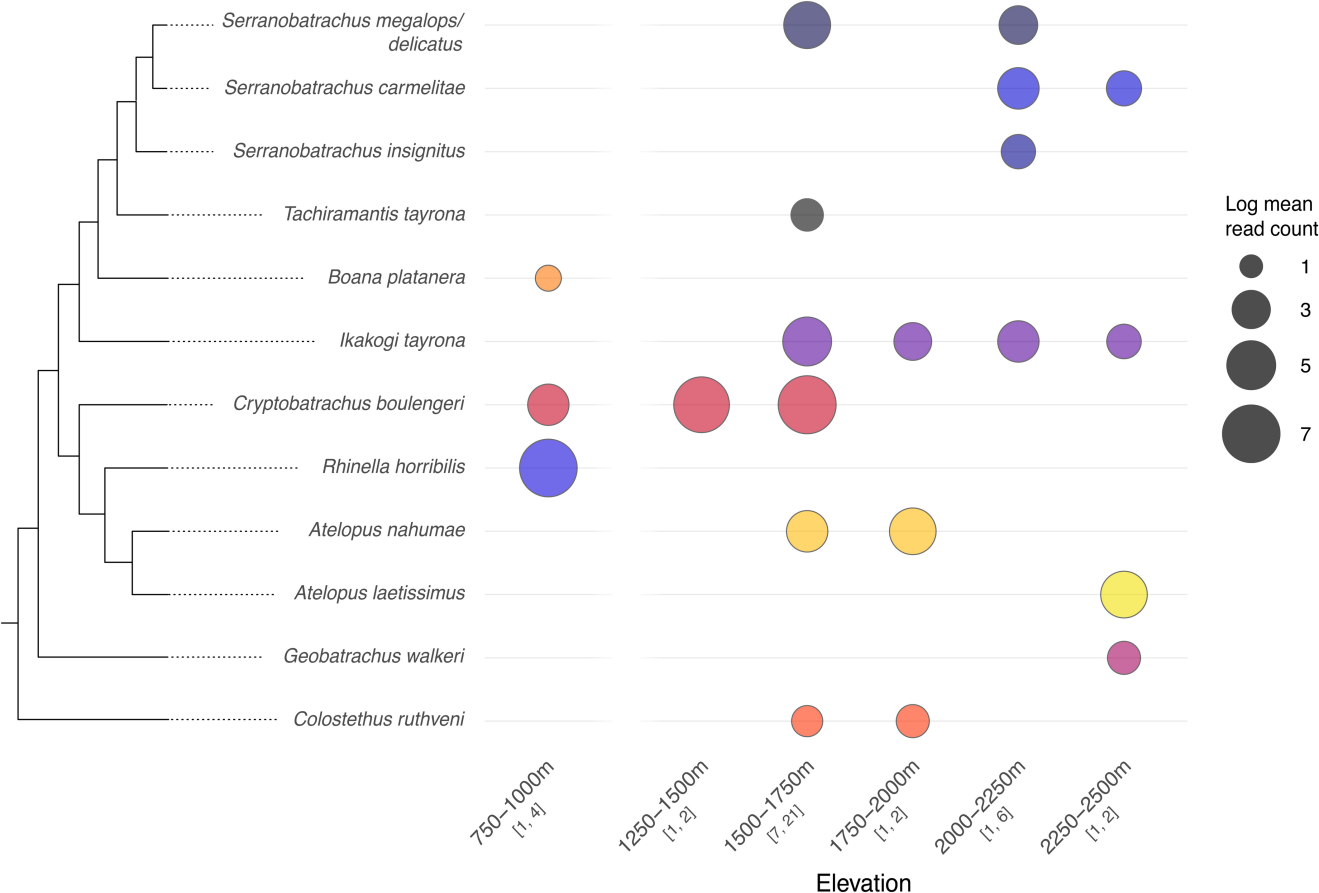
Environmental DNA (eDNA) sequencing read counts for all species that were correctly assigned to species (i.e., species visually observed and identified) across elevations in the Sierra Nevada de Santa Marta in northern Colombia. The data are presented as the natural logarithm of the mean read count across all replicates in each elevational cohort. The number of sampling sites (first value) and total sequenced field and RPA replicates (second value) per elevational category are indicated in brackets. Note that no sampling sites were situated between 1000–1250 m, and eDNA sampling comprised a non‐homogenous spread across elevations. Colours correspond to species as in Figure 2. Maximum likelihood phylogeny as derived from single‐specimen barcoding (Figure S3).

Our eDNA results confirmed an elevational structure in species distributions, with the lowland generalist species *Rhinella horribilis* and *Boana platanera* found only in the lowest site, and the montane endemics *Atelopus laetissimus, Geobatrachus walkeri* and *Serranobatrachus carmelitae* found only in samples above 2000 m elevation (Figure 3). Species detection from eDNA was more consistent for species with aquatic larval stages (the genera *Atelopus, Boana, Colostethus*, *Ikakogi* and *Rhinella*) or stream‐associated occurrence ( *Cryptobatrachus boulengeri* and *S. carmelitae*), showing no large spatial gaps between sampling sites in their elevational range. These patterns were reflected by absolute read counts, with stream‐breeding and associated species presenting the vast majority of assigned reads from water eDNA samples across our study sites (Figure 2B; Figure 3). Instead, detection of terrestrial or arboreal forest‐associated direct‐developers (the genera *Geobatrachus*, *Serranobatrachus* [except *S. carmelitae*] and *Tachiramantis*) was more inconsistent considering the elevational range in which they have directly been observed (Figure 3; Table S5). Four anuran species (*Serranobatrachus sanctaemartae*, *S. cristinae*, *Lithobates vaillanti* and an undescribed *Tachiramantis* sp.) that were observed directly and included in single‐specimen barcoding were not recovered in any of the eDNA samples. With the exception of *L. vaillanti* , these taxa are similarly associated with a terrestrial and/or arboreal life history (Table S5).

## 4 Discussion

With precipitous and ongoing global biodiversity declines presenting one of the most urgent challenges of our time, leveraging the use of molecular species monitoring tools becomes increasingly important to enable timely conservation action (Altermatt et al. 2025). We here introduce a simplified RPA‐ and Nanopore MinION‐based workflow that uses non‐invasive barcoding of species lacking molecular reference data, and integrate these with existing sequence data to create a highly accurate reference database for community metabarcoding for direct use in the field. Focusing on a threatened and remote tropical amphibian assemblage in northern Colombia, we demonstrated that de novo reference assembly strongly increased the taxonomic accuracy of metabarcoding assessments, doubled the number of visually confirmed species detected in eDNA samples, and increased the proportion of correct species‐level assignments of eDNA sequence reads from 12.2% to 93.1% at a 95% sequence identity cutoff. At a more stringent 99% sequence similarity cutoff, correct species‐level assignment rose from ~50% to near‐complete accuracy. Two out of 12 correctly identified species were nevertheless recovered below the stringent 99% cutoff, emphasising that a higher threshold involves a trade‐off with the number of identified species, and a robust interpretation of results requires optimisation across different study systems.

In turn, employing our two‐step barcoding assay enabled a better characterisation of the elevational community structure of amphibian species. However, as commonly observed for eDNA from water samples, detection was more consistent for species with aquatic larvae or stream‐associated life histories than for arboreal or terrestrial species. Several, mostly terrestrial species present in the combined (de novo assembled) reference database were not detected in eDNA samples, reflecting the life history of species with a reduced likelihood of shedding genetic material into aquatic systems (de Souza et al. 2016; Ruppert et al. 2019; Davis et al. 2025).

De novo reference sequence generation not only enhanced resolution but also strongly increased the number of reads passing the similarity thresholds and being assigned to target taxa, implying that a lower sequencing depth may be sufficient for confident assessments. This is a critical consideration when operating with limited flow cell capacity (such as the Flongle flow cell used in this study) and time constraints in remote field settings (De La Cerda et al. 2023, *cf*. Zorz et al. 2023). Further advances in taxonomic resolution could be achieved by targeting longer markers for metabarcoding, as increasingly feasible with third‐generation sequencing (Heeger et al. 2018; Jamy et al. 2020; Ohta et al. 2023; Plewnia, Krehenwinkel, and Heine 2025; Ip et al. 2026). For environmental samples, however, marker length is constrained by DNA quality (Brandão‐Dias et al. 2025), the likely cause for only short markers consistently amplifying environmental samples with RPA in our dataset. Indeed, long markers may ‘overlook’ rare species in environmental samples where DNA fragmentation prevents amplification (Doorenspleet et al. 2025). A solution for this could be the use of several, stepwise nested markers, where complete community composition is approximated by a short marker, nested within a longer target fragment that allows increased taxonomic resolution for more abundant species where detection of less degraded DNA (i.e., allowing long‐read amplification) is more likely (Stoeckle et al. 2018; Ruppert et al. 2019; Brandão‐Dias et al. 2025). Our final primer selection represents a fraction of all possible combinations and, in addition, we could not exhaustively test the functionality of all primer pairs. Optimising and scaling our approach will strongly benefit from a better automated metabarcoding primer design pipeline that takes RPA‐specific requirements such as longer primers and higher mismatch tolerance into account. Several of the RPA primers we designed and utilised for our study successfully amplified DNA from a broad range of amphibian taxa from environmental samples. Furthermore, the primers yielded partial metabarcoding ‘bycatch’, mostly in the form of other vertebrate taxa, which can add valuable information for exhaustive habitat‐scale biodiversity assessments (Plewnia, Hildwein, et al. 2026).

Our workflow can be readily adapted to other taxonomic groups and ecosystems where reference coverage remains poor, thus directly contributing to the establishment of comparative molecular data for regions that are particularly understudied (Marques et al. 2021). Future developments for the field‐deployable eDNA toolkit may explore amplification‐free approaches such as isothermal and non‐invasive CRISPR‐capture strategies (Plewnia, Hoenig, et al. 2026). Coupled with MinION sequencing, this could allow simultaneous species detection and methylation profiling from ‘native’ DNA. Such methods hold promise for adding demographic and health indicators to biodiversity assessments (e.g., Crossman et al. 2021; Zhao et al. 2023; Le Clercq et al. 2023; Anderson et al. 2024). Further improvements to field‐based metabarcoding assays may include affinity‐tagged primers which, in combination with lateral flow detection, could allow visualisation of amplification success prior to pooling and sequencing on site with minimal additional equipment (Lobato and O'Sullivan 2018; Plewnia, Hoenig, et al. 2026).

In summary, by integrating single‐specimen reference generation directly into a PCR‐free field‐deployable metabarcoding workflow, we can bridge critical data gaps that are currently impeding accurate molecular biodiversity monitoring in high‐diversity but understudied ecosystems. Our approach is portable, rapid and scalable, providing practitioners in remote and resource‐limited settings with the capacity to generate high‐quality, community‐level biodiversity data, with environmental sampling and species‐specific sampling being carried out in parallel. Applying this workflow in biodiversity hotspots can accelerate the creation of essential ecological baseline data and strengthen conservation decision‐making in the very regions where timely action is most urgently needed.

## Author Contributions

J.E., A.P. and C.H. initiated the idea and conceptualised the study. J.E. conducted lab work with contributions from A.P. and C.H. J.E. and A.P. analysed and visualised the data with contributions from C.H. A.J.C. and L.A.R.‐S. helped with sample and permit acquisition. S.L. and H.K. provided project resources and administration. J.E. and A.P. wrote the first draft. All authors revised and edited the manuscript.

## Funding

This work was supported by a postdoctoral startup grant from Trier University through the Forschungsinitiative Rheinland‐Pfalz under number FI‐NF 2025‐05 and 2025‐13, and partially funded under the Biodiversitätsmonitoring 2.0 project by the Ministerium für Wirtschaft, Verkehr, Landwirtschaft und Weinbau Rheinland‐Pfalz. Sample collection was partially funded by the Deutsche Gesellschaft für Herpetologie und Terrarienkunde and the Forschungsfonds of Trier University.

## Ethics Statement

All research was carried out using non‐invasive methods. Samples used in this study were collected and exported under permits P02182S9571_N0055 and Permiso no CITES 003790 granted by Autoridad Nacional de Licencias Ambientales, ANLA, Colombia.

## Conflicts of Interest

The authors declare no conflicts of interest.

## Supporting information


**Data S1:** men70122‐sup‐0001‐supinfo.pdf.

## Data Availability

All data underlying this paper are provided in the Supporting Information, and additional supplementary data (demultiplexed MinION raw reads and read count tables) can be found on the Figshare open‐access repository at https://doi.org/10.6084/m9.figshare.28417451. The generated 16S reference sequences are available on GenBank under accession numbers PX105574–PX105645 (see Table S2 for details).
