# Supplementary material for: A Field‐Deployable eDNA Metabarcoding Workflow Including De Novo Reference Assembly for Characterising Understudied Biodiversity Hotspots
Source: Mol Ecol Resour. 2026 Mar 31;26(3):e70122. doi: 10.1111/1755-0998.70122 (PMC13036477; doi:10.1111/1755-0998.70122)
Supplement: Supplementary file 1 — Data S1: men70122‐sup‐0001‐supinfo.pdf. [file MEN-26-e70122-s001.pdf]

Supporting Information

A field-deployable eDNA metabarcoding workflow including *de novo* reference assembly for characterising understudied biodiversity hotspots

Jesse Erens[1,2,3,*], Christopher Heine[1], Stefan Lötters[1,2], Henrik Krehenwinkel[1], Andrew J. Crawford[4], Luis Alberto Rueda-Solano[5], Amadeus Plewnia[1,2]

[1] Biogeography Department, Trier University, Trier, Germany

[2] Habitats Conservation, Hostert, Luxembourg

[3] Marine Evolution and Ecology group, Naturalis Biodiversity Center, Leiden, The Netherlands

[4] Department of Biological Sciences, Universidad de los Andes, Bogotá, Colombia

[5] Facultad de Ciencias Básicas, Universidad del Magdalena, Santa Marta, Colombia

[*] Corresponding author: jesse.erens@outlook.com

Molecular Ecology Resources (2026)

https://doi.org/10.1111/1755-0998.70122

Table S1. List of primers tested for use with Recombinase Polymerase Amplification (RPA). Bases that were edited or added with respect to previously published primers (see references) are highlighted in bold. Primers not adapted from existing references were designed by visually scanning the reference alignment (Portik *et al.* 2023) for conserved regions. For the column 'Site': numbers and colours correspond to the different primer binding sites on the 16S rRNA gene as illustrated in Figure S1. Dots highlight the primer pairs selected for use in final analyses (• Long-read marker for DNA isolates; •• Short-read marker for eDNA samples; also see Table S2).

Primer name	F/R	Sequence (5'-3')	Reference	Site
**MiAmphiL_ext28_F ••**	F	CCTCGCCTGTTTACCAAAAAC**AYCGCCT**	Sakata *et al.* 2022	1
MiAmphiL_ext30_F	F	**R**CCTCGCCTGTTTACCAAAAAC**AYCGCCTY**		
MiA16SF_25ext	F	GCCTGTTTACCAAAAAC**AYCGCCT**		
MiA16SRasF_27ext1	F	**AYWAG**ACGAGAAGACCCCATGGAG**CTT**	Sakata *et al.* 2022 (modified: reverse as forward)	2
MiA16SRasF_27ext2	F	**AYAAG**ACGAGAAGACCCCATGGAG**CTT**		
MiA16SRasF_25ext	F	**AAG**ACGAGAAGACCCCATGGAG**CTT**		
MiA16SRasF_25extD	F	**AAG**ACGAGAAGACCC**Y**ATGGAG**CTT**		
**MiAmphiL_ext_R •**	R	**AAG**CTCCAT**R**GGGTCTTCTCGT**CTWRT**	Sakata *et al.* 2022	3
MiA16SR_27ext1	R	**AAG**CTCCATGGGGTCTTCTCGT**CTWRT**		
MiA16SR_27ext2	R	**AAG**CTCCATGGGGTCTTCTCGT**CTTRT**		
MiA16SR_25ext	R	**AAG**CTCCATGGGGTCTTCTCGT**CTT**		
MiA16SR_25extD	R	**AAG**CTCCAT**R**GGGTCTTCTCGT**CTT**		
16S_APJE_R1	R	GCGCTGTTATCCCYAGGGTAACTTGGTTC	No reference used	4
16S_APJE_R2	R	GCTGTTATCCCYAGGGTAACTTGGTTC		
16S_APJE_R3	R	GCGCTGTTATCCCYAGGGTAACTTGGT		
16S_APJE_R4	R	TGTTATCCCYAGGGTAACTTGGTTC		
16S_preMig_R1	R	CCTGATCCAACATCGAGGTCGTAAACC	No reference used	5
16S_preMig_R2	R	TGATCCAACATCGAGGTCGTAAACC		
16S_preMig_R3	R	CCTGATCCAACATCGAGGTCGTAAACC		
16S_preMig_R4	R	CCTGATCCAACATCGAGGTCGTAAACC		
**16SbH •**	R	CCGGTCTGAACTCAGATCACGT	Palumbi *et al.* 2002	6
16SbH_ext	R	**CY**CCGGTCTGAACTCAGATCACGT**RRGGYWT**		
16SbH_ext_28	R	**CY**CCGGTCTGAACTCAGATCACG**TRRGG**		
16SbH_ext_26	R	CCGGTCTGAACTCAGATCACGT**RRGG**		
16SbH_ext_26nd	R	CCGGTCTGAACTCAGATCACGT**AGGG**		

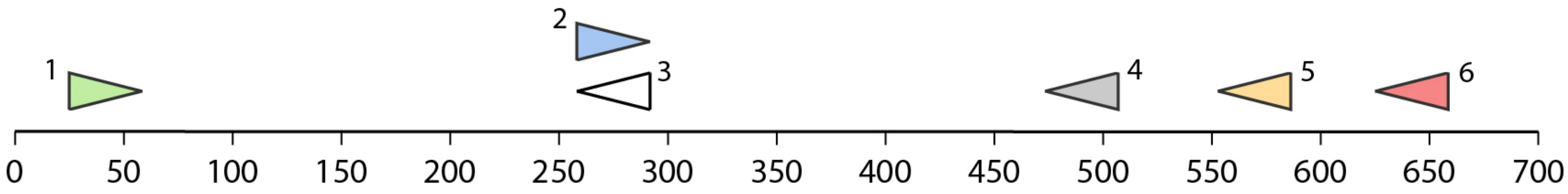

Figure S1. Map of primer binding sites on the 16S rRNA mitochondrial gene. Numbers and colours correspond to the primer overview in Table S1. The positions are in reference to *Xenopus laevis* in the 16S alignment by Portik *et al.* (2023). Note that positions may vary between species.

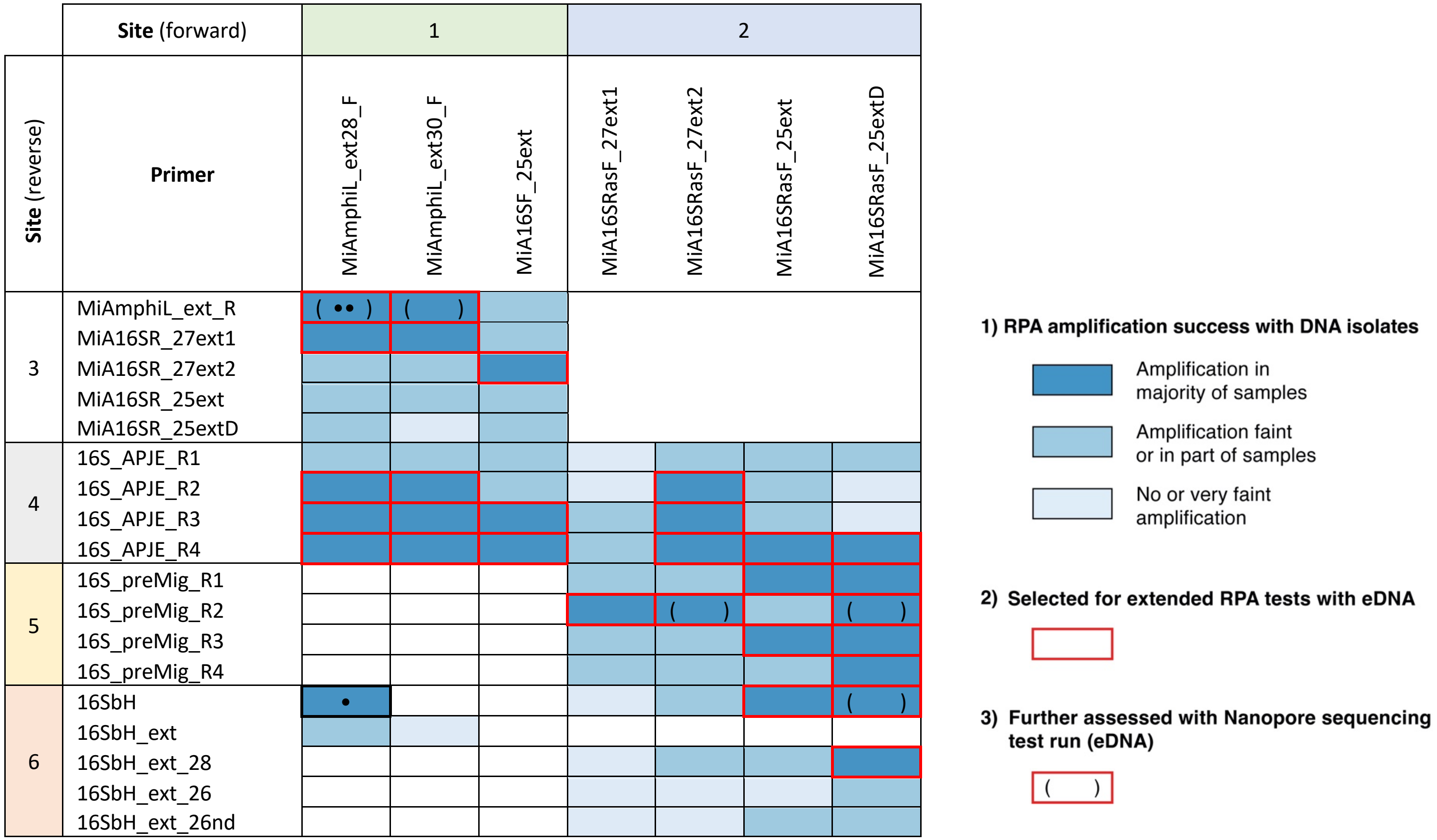

Figure S2. Overview of primer combinations tested for use with Recombinase Polymerase Amplification (RPA). Following RPA tests on DNA isolates from non-invasive amphibian samples (skin swabs), primer combinations showing successful amplification (based on visualization with gel electrophoresis; see legend) were taken for extended testing with environmental DNA (eDNA) samples (outlined in red). Given resource limitations, not all primer combinations could be tested on a broader subset of samples - primer combinations in white could not be tested or only on a single sample, precluding an assessment of their functionality. For eDNA samples, final primer selection was based on a Nanopore sequencing test run. Dots highlight the primer pairs selected for use in final analyses (• Long-read marker for DNA isolates; •• Short-read marker for eDNA samples). For the primer binding sites, numbers and colours correspond to Table S1 and Figure S1.

Table S2. GenBank accession numbers for newly generated 16S sequences from non-invasive skin swabs of amphibian species in the Sierra Nevada de Santa Marta, northern Colombia. Only generalized locality names are provided to protect already threatened populations (Lindenmayer & Scheele, 2017).

**Species**	**Locality**	**GenBank #**
Bolitoglossa savagei	Sogrome	PX105574
Bolitoglossa savagei	San Pedro	PX105575
Bolitoglossa savagei	San Pedro	PX105576
Serranobatrachus megalops/delicatus	Cuchillo San Lorenzo	PX105577
Serranobatrachus megalops/delicatus	Cuchillo San Lorenzo	PX105578
Serranobatrachus megalops/delicatus	Cuchillo San Lorenzo	PX105579
Serranobatrachus megalops/delicatus	Cuchillo San Lorenzo	PX105580
Serranobatrachus megalops/delicatus	Cuchillo San Lorenzo	PX105581
Serranobatrachus megalops/delicatus	San Pedro	PX105582
Serranobatrachus megalops/delicatus	San Pedro	PX105583
Serranobatrachus sanctaemartae	Cuchillo San Lorenzo	PX105584
Serranobatrachus carmelitae	Cuchillo San Lorenzo	PX105585
Serranobatrachus sanctaemartae	San Pedro	PX105586
Serranobatrachus cristinae	Cuchillo San Lorenzo	PX105587
Serranobatrachus cristinae	Cuchillo San Lorenzo	PX105588
Serranobatrachus cristinae	Cuchillo San Lorenzo	PX105589
Serranobatrachus cristinae	Cuchillo San Lorenzo	PX105590
Serranobatrachus insignitus	San Pedro	PX105591
Serranobatrachus insignitus	Cuchillo San Lorenzo	PX105592
Serranobatrachus insignitus	San Pedro	PX105593
Serranobatrachus cf. *carmelitae*	San Pedro	PX105594
Serranobatrachus cf. *carmelitae*	San Pedro	PX105595
Serranobatrachus carmelitae	San Pedro	PX105596
Serranobatrachus carmelitae	San Pedro	PX105597
Tachiramantis tayrona	Cuchillo San Lorenzo	PX105598
Tachiramantis tayrona	Cuchillo San Lorenzo	PX105599
Tachiramantis sp.	Río Tucurinca	PX105600
Tachiramantis sp.	Río Tucurinca	PX105601
Tachiramantis sp.	Cuchillo San Lorenzo	PX105602
Tachiramantis sp.	Río Tucurinca	PX105603
Tachiramantis sp.	Río Tucurinca	PX105604
Tachiramantis sp.	Cuchillo San Lorenzo	PX105605
Tachiramantis sp.	Río Tucurinca	PX105606
Tachiramantis sp.	Cuchillo San Lorenzo	PX105607
Tachiramantis sp.	Cuchillo San Lorenzo	PX105608
Tachiramantis sp.	Cuchillo San Lorenzo	PX105609
Lithobates vaillanti	Palmor	PX105610
Lithobates vaillanti	Palmor	PX105611
Colostethus ruthveni	Palmor	PX105612
Colostethus ruthveni	Río Tucurinca	PX105613
Colostethus ruthveni	Cuchillo San Lorenzo	PX105614
Boana platanera	Palmor	PX105615
Boana platanera	Palmor	PX105616
Boana platanera	Palmor	PX105617
Geobatrachus walkeri	Cuchillo San Lorenzo	PX105618
Geobatrachus walkeri	Cuchillo San Lorenzo	PX105619
Cryptobatrachus boulengeri	Cuchillo San Lorenzo	PX105620
Cryptobatrachus boulengeri	Cuchillo San Lorenzo	PX105621
Cryptobatrachus boulengeri	Cuchillo San Lorenzo	PX105622
Cryptobatrachus boulengeri	Cuchillo San Lorenzo	PX105623
Cryptobatrachus boulengeri	Cuchillo San Lorenzo	PX105624
Cryptobatrachus boulengeri	San Pedro	PX105625
Cryptobatrachus boulengeri	Palmor	PX105626
Cryptobatrachus boulengeri	Río Tucurinca	PX105627
Cryptobatrachus boulengeri	Río Tucurinca	PX105628
Ikakogi tayrona	Cuchillo San Lorenzo	PX105629
Ikakogi tayrona	Cuchillo San Lorenzo	PX105630

Ikakogi tayrona	Cuchillo San Lorenzo	PX105631
Rhinella horribilis	Palmor	PX105632
Rhinella horribilis	Palmor	PX105633
Rhinella horribilis	Palmor	PX105634
Atelopus nahumae	Cuchillo San Lorenzo	PX105635
Atelopus nahumae	Cuchillo San Lorenzo	PX105636
Atelopus nahumae	Cuchillo San Lorenzo	PX105637
Atelopus nahumae	Cuchillo San Lorenzo	PX105638
Atelopus nahumae	Cuchillo San Lorenzo	PX105639
Atelopus laetissimus	San Pedro	PX105640
Atelopus laetissimus	Cuchillo San Lorenzo	PX105641
Atelopus laetissimus	Cuchillo San Lorenzo	PX105642
Atelopus laetissimus	Cuchillo San Lorenzo	PX105643
Atelopus laetissimus	Cuchillo San Lorenzo	PX105644
Atelopus laetissimus	Cuchillo San Lorenzo	PX105645

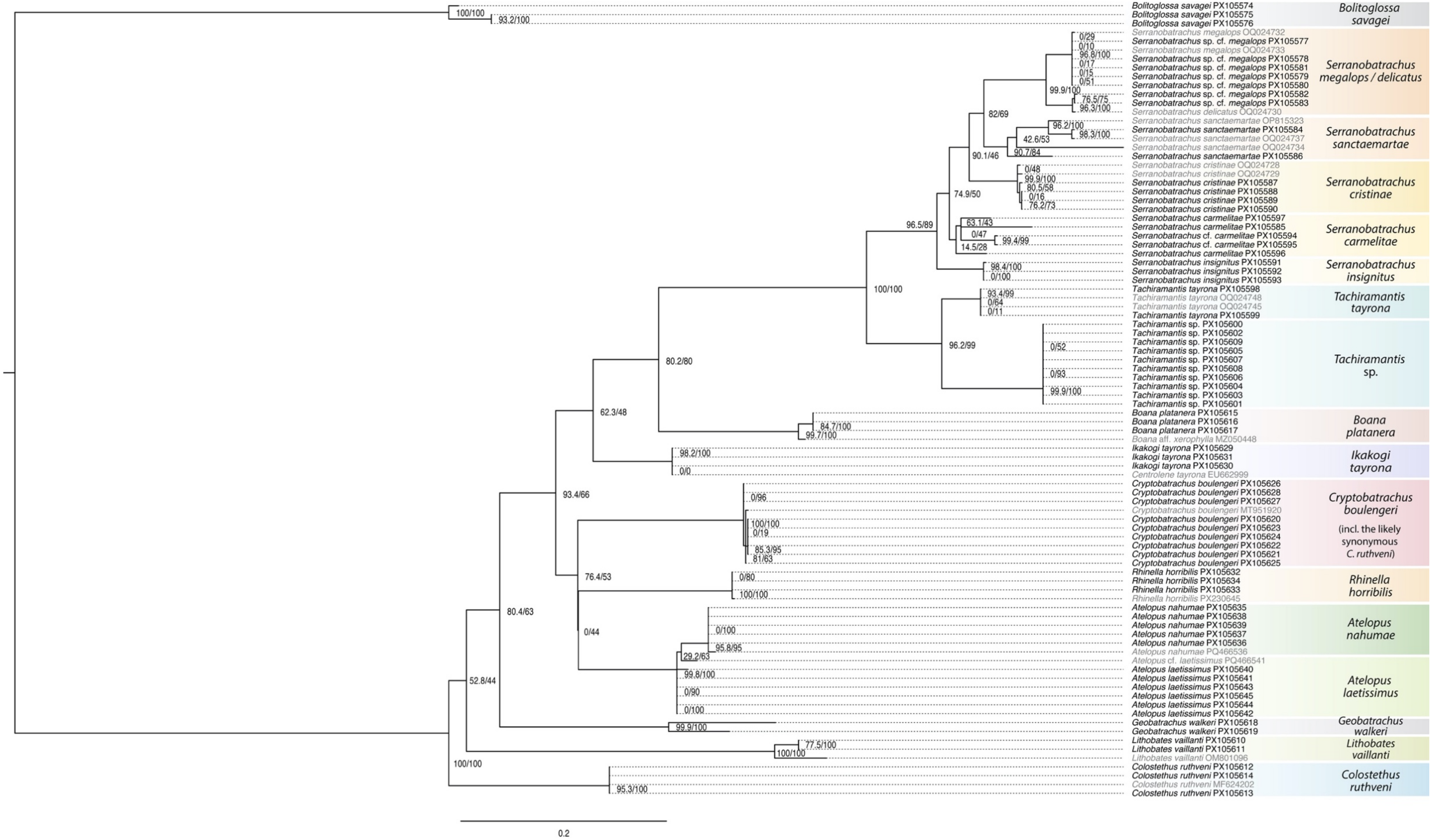

Figure S3. Maximum Likelihood tree inferred from a long-read 16S rRNA alignment (561 bp) of Nanopore consensus sequences derived from 72 non-invasive skin swab samples of species encountered in the Sierra Nevada de Santa Marta in northern Colombia (in black), complemented with available GenBank sequences (in grey). *Bolitoglossa* was used as outgroup. Node values designate ultrafast bootstrap and approximate likelihood ratio test (aLRT) values, respectively, based on 100.000 pseudoreplicates. *Boana platanera* is a recently described species that was previously included as *Boana* aff. *xerophylla* in GenBank and we considered a 'correct' species assignment accordingly. *Ikakogi tayrona* was previously classified under *Centrolene*. It should be noted that the taxonomic status of *Atelopus* cf. *laetissimus* (GenBank: PQ466541) is uncertain and in need of further clarification. Note that intergeneric relationships are poorly supported and phylogenetic estimates are inconsistent when based on 16S barcoding sequences only (see e.g. Chan et al. 2022).

Table S3. Uncorrected pairwise genetic distances (in %) among sequences included in the 16S rRNA alignment (561 bp) used for phylogenetic analyses (Figure S3). Identical sequences were merged to remove duplicate results, with the number of identical sequences being displayed in brackets and in bold. Sequences obtained from GenBank are highlighted in yellow/orange.

Bolitoglossa savagei PX105574	*Bolitoglossa savagei* PX105575-PX105576 **(2)**	*Lithobates vaillanti* PX105610-PX105611 **(2)**	*Lithobates vaillanti* OM801096	*Tachiramantis* sp. PX105600-PX105609 **(10)**	*Tachiramantis tayrona* OQ024745 + OQ024748 **(2)**	*Tachiramantis tayrona* PX105598-PX105599 **(2)**	*Serranobatrachus* sp. cf. *megalops* PX105577-PX105581 (	*Serranobatrachus megalops* OQ024732-OQ024733 **(2)**	*Serranobatrachus* sp. cf. *megalops* PX105582	*Serranobatrachus delicatus* OQ024730	*Serranobatrachus* sp. cf. *megalops* PX105583	*Serranobatrachus sanctaemartae* PX105586	*Serranobatrachus sanctaemartae* OP815323	*Serranobatrachus sanctaemartae* PX105584	*Serranobatrachus sanctaemartae* OQ024737	*Serranobatrachus sanctaemartae* OQ024734	*Serranobatrachus carmelitae* PX105585	*Serranobatrachus cristinae* OQ024728	*Serranobatrachus cristinae* OQ024729	*Serranobatrachus cristinae* PX105587	*Serranobatrachus cristinae* PX105588-PX105590 **(3)**	*Serranobatrachus insignitus* PX105591	*Serranobatrachus insignitus* PX105592-PX105593 **(2)**	*Serranobatrachus* cf. *carmelitae* PX105594	*Serranobatrachus* cf. *carmelitae* PX105595	*Serranobatrachus carmelitae* PX105596	*Serranobatrachus carmelitae* PX105597	*Colostethus ruthveni* PX105612-PX105614 **(3)**	*Colostethus ruthveni* MF624202	*Geobatrachus walkeri* PX105618	*Geobatrachus walkeri* PX105619	*Boana platanera* PX105615-PX105617 **(3)**	*Boana* aff. *xerophylla* MZ050448	*Cryptobatrachus boulengeri* MT951920	*Cryptobatrachus boulengeri* PX105620-PX105624 **(5)**	*Cryptobatrachus boulengeri* PX105626-PX105628 **(3)**	*Cryptobatrachus boulengeri* PX105625	*Rhinella horribilis* PX105632-PX105634 **(3)**	*Rhinella horribilis* PX230645	*Ikakogi tayrona* PX105629-PX105631 **(3)**	*Centrolene tayrona* EU662999	*Atelopus nahumae* PX105635-PX105639 **(5)**	*Atelopus nahumae* PQ466536	*Atelopus* cf. *laetissimus* PQ466541	*Atelopus laetissimus* PX105640	*Atelopus laetissimus* PX105641-PX105645 **(5)**	
0.00	3.27	25.71	26.29	24.85	26.11	25.05	25.82	27.02	24.95	24.95	24.95	25.61	26.29	24.95	25.06	27.25	24.90	27.41	24.45	23.67	23.47	26.12	25.92	24.69	25.10	25.31	25.71	24.64	24.64	24.90	23.83	24.03	24.24	24.18	24.03	24.03	24.03	25.05	25.25	24.03	24.42	24.24	25.25	25.50	24.18	23.83	*Bolitoglossa savagei* PX105574
	0.00	26.48	27.74	25.82	27.37	26.23	26.58	27.83	26.12	26.12	26.12	26.38	27.10	25.71	26.12	27.40	25.66	28.78	25.27	24.44	24.24	26.48	26.27	25.66	26.07	26.07	26.48	26.83	26.83	25.36	23.98	25.41	25.61	25.65	25.41	25.41	25.41	26.42	26.63	25.61	26.05	25.00	26.42	26.17	24.95	24.59	*Bolitoglossa savagei* PX105575-PX105576 **(2)**
		0.00	5.56	22.10	22.70	21.37	23.43	24.60	23.34	23.34	23.34	23.76	25.30	23.91	25.36	23.57	22.35	29.13	24.80	23.11	23.11	22.16	21.97	21.59	21.78	22.35	23.30	18.49	18.49	21.04	19.27	19.13	18.98	19.96	19.92	19.92	19.92	19.32	19.66	18.87	19.46	19.47	22.62	22.62	19.77	19.09	*Lithobates vaillanti* PX105610-PX105611 **(2)**
			0.00	23.95	24.73	23.05	25.00	26.48	25.25	25.25	25.25	25.50	27.41	25.45	27.55	25.10	23.21	31.65	26.71	24.60	24.80	22.82	22.62	23.02	23.21	23.21	24.21	20.75	20.75	22.67	21.21	21.23	20.87	20.88	21.07	21.07	21.07	18.56	19.05	20.36	21.02	20.40	22.97	22.52	20.72	20.00	*Lithobates vaillanti* OM801096
				0.00	9.15	8.57	15.86	16.73	15.21	15.40	15.03	15.80	16.15	16.51	17.30	17.47	16.11	19.85	16.47	15.19	15.19	15.00	14.81	15.74	15.93	15.19	14.81	22.00	22.00	22.56	21.56	20.59	20.41	21.60	21.28	21.28	21.28	21.93	22.55	19.78	20.38	20.33	23.01	23.06	20.67	20.19	*Tachiramantis* sp. PX105600-PX105609 **(10)**
					0.00	0.00	15.74	15.71	14.88	14.68	14.68	13.69	14.09	14.29	14.49	15.08	14.88	16.84	13.92	13.69	13.69	13.69	13.49	12.50	12.70	13.49	13.10	23.17	23.17	23.57	21.68	19.09	19.48	21.12	20.61	20.61	20.61	21.51	21.78	20.40	20.40	21.39	22.39	22.22	20.56	20.44	*Tachiramantis tayrona* OQ024745 + OQ024748 **(2)**
						0.00	14.71	15.55	13.75	13.75	13.57	12.83	14.06	13.75	14.52	14.31	13.75	16.62	13.75	12.64	12.64	12.64	12.45	11.71	11.90	12.64	12.08	21.89	21.89	22.22	20.46	18.06	18.44	20.08	19.66	19.66	19.66	20.48	20.78	19.48	19.89	20.22	22.22	22.05	19.63	19.33	*Tachiramantis tayrona* PX105598-PX105599 **(2)**
							0.00	0.00	3.88	4.05	3.70	8.69	9.86	11.09	11.60	12.20	10.54	11.87	10.47	9.58	9.58	9.80	9.61	9.06	9.06	8.69	8.50	22.92	22.92	23.56	22.56	20.59	21.11	21.08	20.19	20.19	20.19	21.97	22.08	19.96	20.38	20.93	23.33	23.61	21.64	21.34	*Serranobatrachus* sp. cf. *megalops* PX105577-PX105581 **(5)**
								0.00	4.29	4.27	4.09	9.36	10.08	11.50	11.58	12.87	11.31	12.31	10.65	10.29	10.29	10.53	10.33	9.55	9.55	9.16	9.16	23.98	23.98	24.70	23.64	21.53	22.07	21.91	20.92	20.92	20.92	22.83	22.90	20.86	20.86	21.88	23.50	23.78	22.44	22.31	*Serranobatrachus megalops* OQ024732-OQ024733 **(2)**
									0.00	0.55	0.18	9.21	10.23	11.03	11.55	11.97	10.66	12.85	11.02	9.93	9.93	9.93	9.74	9.19	9.19	8.82	9.01	23.35	23.35	23.28	22.10	21.22	20.85	21.36	20.45	20.45	20.45	22.08	22.32	20.22	20.64	20.99	23.18	22.57	20.96	20.66	*Serranobatrachus* sp. cf. *megalops* PX105582
										0.00	0.37	9.39	10.19	10.85	11.33	12.15	10.66	12.78	11.00	10.26	10.26	9.93	9.74	9.19	9.19	8.64	9.01	23.35	23.35	23.43	22.10	21.03	20.66	21.56	20.64	20.64	20.64	21.89	21.96	20.04	20.45	20.81	22.96	22.35	20.78	20.48	*Serranobatrachus delicatus* OQ024730
											0.00	9.02	10.04	10.85	11.35	11.79	10.48	12.59	10.83	9.74	9.74	9.74	9.56	9.01	9.01	8.64	8.82	23.16	23.16	23.28	22.10	21.03	20.66	21.36	20.45	20.45	20.45	21.89	22.14	20.04	20.45	20.81	22.96	22.35	20.78	20.48	*Serranobatrachus* sp. cf. *megalops* PX105583
												0.00	6.95	7.55	7.98	9.58	10.85	9.07	8.46	7.72	7.72	9.93	9.74	8.82	8.82	7.90	7.17	22.47	22.47	23.52	21.18	18.85	18.67	20.40	19.55	19.55	19.55	20.82	21.07	19.34	19.73	20.66	22.12	21.51	20.45	20.15	*Serranobatrachus sanctaemartae* PX105586
													0.00	3.28	3.38	9.67	11.92	10.20	9.20	9.00	9.00	11.35	11.15	10.38	10.38	9.04	9.04	24.86	24.86	25.00	22.40	20.12	19.92	22.69	21.65	21.65	21.65	23.15	23.21	20.04	20.04	22.59	23.35	23.18	21.79	21.86	*Serranobatrachus sanctaemartae* OP815323
														0.00	0.40	10.50	11.76	9.82	9.25	9.01	9.01	11.76	11.58	10.85	10.85	9.19	9.19	24.63	24.63	24.05	22.67	19.00	19.37	21.76	20.83	20.83	20.83	22.26	22.32	19.67	20.27	22.10	23.84	23.67	21.52	21.40	*Serranobatrachus sanctaemartae* PX105584
															0.00	10.98	12.35	9.80	9.16	9.15	9.15	12.35	12.15	11.16	11.16	9.56	9.16	26.10	26.10	25.47	23.60	20.20	20.60	23.09	22.00	22.00	22.00	23.54	23.80	20.72	20.72	23.55	24.22	24.05	22.74	22.80	*Serranobatrachus sanctaemartae* OQ024737
																0.00	11.79	12.37	11.64	10.87	10.87	11.79	11.60	10.13	10.50	9.94	8.66	23.57	23.57	22.75	20.99	20.89	20.70	22.60	21.62	21.62	21.62	21.93	22.18	21.18	21.63	22.88	24.78	23.95	22.49	22.37	*Serranobatrachus sanctaemartae* OQ024734
																	0.00	13.03	10.59	9.52	9.89	8.42	8.24	6.96	6.96	7.14	7.14	21.47	21.47	22.86	21.10	18.78	18.78	19.52	18.73	18.73	18.73	21.85	22.10	18.17	18.53	20.77	23.13	22.08	20.93	20.63	*Serranobatrachus carmelitae* PX105585
																		0.00	0.50	0.75	0.75	11.53	11.28	8.52	9.02	9.52	10.28	28.39	28.39	26.91	24.01	23.23	23.23	26.48	24.81	24.81	24.81	26.72	26.77	24.87	24.87	25.44	26.88	26.96	25.45	25.51	*Serranobatrachus cristinae* OQ024728
																			0.00	0.78	0.39	9.41	9.22	7.06	7.45	7.84	9.02	24.17	24.17	24.08	21.43	20.12	20.12	22.32	21.29	21.29	21.29	22.62	22.88	21.41	21.41	21.85	22.52	22.57	21.83	21.89	*Serranobatrachus cristinae* OQ024729
																				0.00	0.36	8.61	8.42	6.59	6.96	6.96	8.24	22.75	22.75	23.00	20.15	19.15	18.97	21.12	20.22	20.22	20.22	21.48	21.73	20.55	20.98	21.14	22.91	22.74	21.11	20.99	*Serranobatrachus cristinae* PX105587
																					0.00	8.79	8.61	6.78	7.14	7.14	8.42	22.75	22.75	22.62	20.15	19.15	19.15	21.31	20.41	20.41	20.41	21.30	21.55	20.55	20.98	20.77	22.47	22.52	20.93	20.81	*Serranobatrachus cristinae* PX105588-PX105590 **(3)**
																						0.00	0.18	7.51	7.69	6.41	6.96	23.30	23.30	23.24	21.10	19.71	19.15	21.51	20.60	20.60	20.60	21.48	21.73	19.45	19.85	21.32	23.57	22.30	21.30	20.99	*Serranobatrachus insignitus* PX105591
																							0.00	7.33	7.51	6.23	6.78	23.30	23.30	23.05	20.91	19.52	18.97	21.31	20.41	20.41	20.41	21.30	21.55	19.27	19.66	21.14	23.35	22.08	21.11	20.81	*Serranobatrachus insignitus* PX105592-PX105593 **(2)**
																								0.00	0.37	5.49	4.95	21.83	21.83	22.29	21.29	18.05	17.86	20.32	19.48	19.48	19.48	21.48	21.73	18.72	19.09	21.69	23.79	21.63	20.74	20.63	*Serranobatrachus* cf. *carmelitae* PX105594
																									0.00	5.49	4.95	21.83	21.83	22.48	21.48	18.23	18.05	20.52	19.66	19.66	19.66	21.67	21.92	18.90	19.28	21.88	24.01	21.85	20.93	20.81	*Serranobatrachus* cf. *carmelitae* PX105595
																										0.00	4.58	23.49	23.49	23.05	21.10	19.15	18.60	21.51	20.60	20.60	20.60	21.67	21.73	19.45	19.85	21.32	23.13	22.08	20.93	20.81	*Serranobatrachus carmelitae* PX105596
																											0.00	23.12	23.12	23.05	20.53	19.52	19.15	20.12	19.29	19.29	19.29	21.67	22.10	18.53	18.90	21.51	23.35	22.08	21.11	20.99	*Serranobatrachus carmelitae* PX105597
																												0.00	0.00	19.54	17.80	17.43	17.61	17.46	16.98	17.35	17.16	17.68	18.13	16.97	17.48	16.82	19.47	18.64	17.31	16.30	*Colostethus ruthveni* PX105612-PX105614 **(3)**
																													0.00	19.54	17.80	17.43	17.61	17.46	16.98	17.35	17.16	17.68	18.13	16.97	17.48	16.82	19.47	18.64	17.31	16.30	*Colostethus ruthveni* MF624202
																														0.00	9.96	19.62	19.62	19.60	19.17	19.17	19.17	18.70	19.17	16.89	17.42	16.89	17.62	18.08	17.75	17.08	*Geobatrachus walkeri* PX105618
																															0.00	19.39	19.17	18.15	17.80	17.61	17.80	16.76	17.23	17.05	17.58	16.86	17.77	17.77	16.57	15.91	*Geobatrachus walkeri* PX105619
																																0.00	1.82	17.13	17.04	16.85	17.04	16.64	17.10	14.44	14.88	16.30	17.98	18.46	16.42	15.78	*Boana platanera* PX105615-PX105617 **(3)**
																																	0.00	17.93	17.79	17.60	17.79	16.08	16.54	14.08	14.50	17.03	18.86	18.02	16.42	15.78	*Boana* aff. *xerophylla* MZ050448
																																		0.00	0.40	0.40	0.20	16.20	16.70	14.09	14.34	14.09	15.25	15.02	13.77	13.10	*Cryptobatrachus boulengeri* MT951920
																																			0.00	0.37	0.19	15.79	16.26	13.43	13.65	14.55	15.47	15.25	14.26	13.62	*Cryptobatrachus boulengeri* PX105620-PX105624 **(5)**
																																				0.00	0.19	15.60	16.07	13.06	13.27	14.37	15.25	15.02	14.07	13.43	*Cryptobatrachus boulengeri* PX105626-PX105628 **(3)**
																																					0.00	15.79	16.26	13.25	13.46	14.55	15.47	15.25	14.26	13.62	*Cryptobatrachus boulengeri* PX105625
																																						0.00	0.18	13.81	14.23	13.08	14.13	14.38	13.73	12.73	*Rhinella horribilis* PX105632-PX105634 **(3)**
																																							0.00	14.10	14.53	13.55	14.69	14.95	14.21	13.21	*Rhinella horribilis* PX230645
																																								0.00	0.00	12.80	14.00	13.82	13.44	12.45	*Ikakogi tayrona* PX105629-PX105631 **(3)**
																																									0.00	13.18	14.00	13.82	13.85	12.83	*Centrolene tayrona* EU662999
																																										0.00	0.66	3.28	3.49	2.55	*Atelopus nahumae* PX105635-PX105639 **(5)**
																																											0.00	3.06	3.72	3.06	*Atelopus nahumae* PQ466536
																																												0.00	2.41	1.75	*Atelopus* cf. *laetissimus* PQ466541
																																													0.00	0.92	*Atelopus laetissimus* PX105640
																																														0.00	*Atelopus laetissimus* PX105641-PX105645 **(5)**

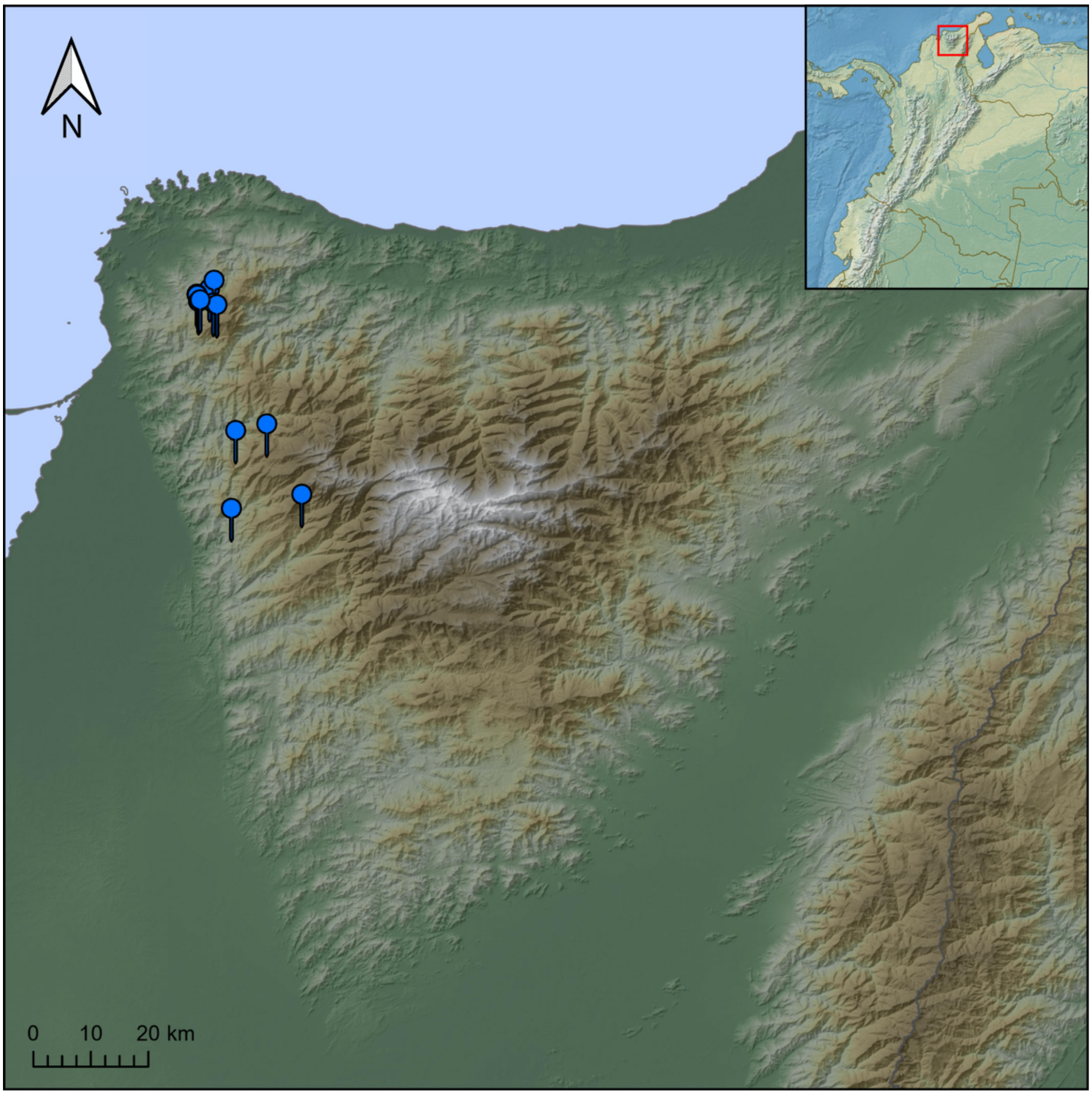

Figure S4. Map of eDNA sampling sites in the Sierra Nevada de Santa Marta (n = 13), with the inset showing its location within Colombia. Basemap adapted from Esri. Inset adapted from Natural Earth (naturalearthdata.com).

Table S4. Overview of eDNA sampling sites with the total number of field sampling and RPA replicates as included in the final Nanopore (MinION) sequencing run. For site 13, no accurate elevation is available.

Site	Elevation	Locality	eDNA samples	RPA replicates	Total sequenced
1	967	Palmor	2	2	4
2	1486	Cuchillo San Lorenzo	1	2	2
3	1505	Cuchillo San Lorenzo	1	2	2
4	1524	Cuchillo San Lorenzo	1	2	2
5	1542	Cuchillo San Lorenzo	1	2	2
6	1550	Cuchillo San Lorenzo	1	2	2
7	1557	San Pedro	2	1 + 2	3
8	1577	Río Tucurinca	2	2	4
9	1667	Cuchillo San Lorenzo	3	2	6
10	1766	Cuchillo San Lorenzo	1	2	2
11	2111	Cuchillo San Lorenzo	3	2	6
12	2459	San Pedro	1	2	2
13	NA	Cuchillo San Lorenzo	1	2	2

Table S5. Anuran species visually observed in the Sierra Nevada de Santa Marta (SNSM) around each respective eDNA sampling locality, including present knowledge on species endemism, habitat characteristics and reproductive mode. Question marks indicate likely presence on the basis of acoustic observations or by proximity to known records. For site 13, no accurate elevation is available.

	Site															
**Species**	**1**	**2**	**3**	**4**	**5**	**6**	**7**	**8**	**9**	**10**	**11**	**12**	**13**	**Endemism**	**Habitat type**	**Reproduction**
Atelopus nahumae	N	N	N	N	N	N	N	Y	N	Y	Y	N	N	SNSM endemic	Riparian (vegetation)	Aquatic larvae (lotic)
Atelopus laetissimus	N	N	N	N	N	N	N	N	N	N	Y	Y	N		Riparian (vegetation)	Aquatic larvae (lotic)
Ikakogi tayrona	N	Y	Y	Y	Y	Y	Y	Y	Y	Y	Y	Y	Y		Riparian (vegetation)	Aquatic larvae (lotic)
Colostethus ruthveni	Y	?	?	?	?	?	Y	Y	?	?	N	N	?		Terrestrial	Aquatic larvae (lentic)
Cryptobatrachus boulengeri (incl. the likely synonymous *C. ruthveni*)	Y	Y	Y	Y	Y	Y	Y	Y	Y	Y	N	N	Y		Riparian (boulders)	Direct development
Tachiramantis tayrona	N	N	N	N	N	N	Y	Y	N	?	Y	Y	N		Bromelicolous	Direct development
Tachiramantis sp.	N	Y	Y	Y	Y	Y	?	Y	N	N	N	N	Y		Lower vegetation	Direct development
Serranobatrachus carmelitae	N	N	N	N	N	N	N	N	N	N	Y	Y	N		Riparian (boulders)	Direct development
Serranobatrachus cristinae	N	N	N	N	N	N	N	N	N	?	Y	?	N		Arboreal	Direct development
Serranobatrachus insignitus	N	N	N	N	N	N	Y	Y	Y	Y	Y	Y	N		Terrestrial	Direct development
Serranobatrachus megalops/delicatus	N	?	?	?	?	?	Y	Y	Y	Y	Y	Y	?		Leaf litter, Lower vegetation	Direct development
Serranobatrachus sanctaemartae	N	Y	Y	Y	Y	Y	Y	Y	Y	Y	Y	Y	Y		Arboreal, Lower vegetation	Direct development
Geobatrachus walkeri	N	N	N	N	N	N	N	N	N	N	Y	Y	N		Fossorial	Direct development
Rhinella horribilis	Y	N	N	N	N	N	N	N	N	N	N	N	N	Lowland species	Terrestrial	Aquatic larvae (lotic + lentic)
Boana platanera	Y	N	N	N	N	N	N	N	N	N	N	N	N		Arboreal	Aquatic larvae (lentic)
Lithobates vaillanti	Y	N	N	N	N	N	N	N	N	N	N	N	N		Terrestrial, Semiaquatic	Aquatic larvae (lentic)
**Elevation**	967	1486	1505	1524	1542	1550	1557	1577	1667	1766	2111	2459	NA			

Protocol S1. Considerations for RPA primer design for metabarcoding.

Given its relatively recent development, existing tools for RPA primer design, such as PrimedRPA (Higgins *et al.* 2019), are largely optimized for finding primers for single target species amplification. Metabarcoding primer design is more challenging, as the primer binding site needs to be conserved over a large range of taxa, while mismatches to non-target taxa need to be maximised. In contrast to PCR, RPA can still yield amplification with several mismatches, challenging the design of specific metabarcoding primers. While for RPA, 30-35 bp primers have been recommended, this might often require the incorporation of several degenerate bases. The latter, however, can easily lead to artefact formation and off-target amplification. Until more robust and automated tools become available, we therefore recommend manual alignment-guided design of several primer combinations and subsequent experimental validation.

A simple approach to RPA metabarcoding primer design is mapping existing PCR metabarcoding primers on a target species alignment and manually extending the primer length towards one or both ends trying to minimize number of degenerate bases. Particularly at the distal positions of the primer, degenerate bases should be avoided to preclude the formation of longer fragments which cannot be easily removed with a magnetic bead clean-up. We observed that for metabarcoding, primers as short as ~22 bp in length (e.g. 16Sbr) can show good amplification in RPA. To assess amplification success during primer design, RPA products are best cleaned with magnetic beads prior to visualization as the RPA's crowding agent can prevent proper flow during gel electrophoresis. We recommend diluting RPA products 1:1 with water prior to magnetic bead clean-up as, similarly, the high density of RPA's crowding agent can prevent beads from rapidly collecting at the magnet.

Protocol S2. Customized protocol for field-deployable library preparation for Flongle sequencing using the SQK-LSK114 kit. RPA-based library preparation follows the supplementary protocol of Plewnia et al. (2025). Indexed RPA-products are pooled to equal amounts. Abbreviations as of SQK-LSK114 kit instructions. Mix all reagents by pipetting up and down.

1. Clean pool with magnetic beads following your manufacturer's instructions for amplicons. Generally, we recommend diluting the pool 1:1 with RNAse-free water before adding beads to prevent the RPA crowding agent from disturbing bead movement to the tube wall.
2. Measure DNA concentration using a Qubit fluorometer following the manufacturer's instructions.
3. Prepare 150-200 ng DNA for library preparation. Normalize to 24.5 µl with RNAse-free water in a PCR strip.
4. Add 0.5 µl DCS, 3.5 µl Ultra II End-prep reaction buffer and 1.5 µl Ultra II End-prep enzyme mix to your input DNA, then mix by pipetting.
5. Incubate for 30 min at ~20°C. This can be done at room temperature or (in cold environments) using a portable heating device (e.g. a 3D-printed heat bed; Plewnia et al. 2025b).
6. Incubate for 30 min at 65°C using a field-deployable heat bed.
7. Add 30 µl of resuspended AMPure XP beads and mix thoroughly by pipetting.
8. Incubate at ambient temperature for 5 min, then place on magnetic rack.
9. When all beads have pelleted on magnet, remove supernatant.
10. Wash by adding and removing 200 µl of 80% ethanol.
11. repeat 10.
12. Remove from magnet and let dry until all droplets have disappeared but avoid overdrying (fissures in bead pellet).
13. Resuspend pellet in 31 µl RNAse-free water, mix thoroughly by pipetting and incubate for 5 min.
14. Place in magnet and wait until beads have pelleted.
15. Transfer 30 µl into next well in the PCR strip.
16. Add 2.5 µl LA, 12.5 µl LNB and 5 µl Quick T4 DNA Ligase, then mix by pipetting.
17. Incubate for 10 min at room temperature (if ambient temperature is below 18°C use field-deployable heat-bed).
18. Add 20 µl resuspended AMPure XP beads, mix thoroughly by pipetting.
19. Incubate at ambient temperature for 5 min, then place on magnetic rack.
20. When all beads have pelleted on magnet, remove supernatant.

21. Wash by adding and removing 125 SFB. Make sure all beads have re-pelleted before removal of SFB as they dissolve more easily in the viscous SFB than in ethanol.

22. repeat 21.

23. Remove from magnet and let dry until all droplets have disappeared but avoid over-drying (fissures in bead pellet).

24. Resuspend pellet in 11 µl EB (this will allow two sequencing runs if needed at the cost of a single lib. prep.), mix thoroughly by pipetting and incubate for 10 min at increased temperature (e.g. in hand or heat-bed at 37°C).

25. Place in magnet and wait until beads have pelleted.

26. Transfer 5 µl each to two tubes of your PCR strip. One is intended as a backup, the other for sequencing.

27. Add 15 µl SB and 10 µl LIB to one tube containing 5µl library. Make sure to mix by pipetting right before loading.

28. In a new tube of your PCR strip, mix 117 µl of FCF with 3 µl of FCT by pipetting.

29. Check and prepare flowcell, then load it and start the sequencing run as described by the manufacturer. Load flowcell by turning the pipette plunger to avoid introduction of air. Make sure to thoroughly close all ports of the flowcell to avoid drying during the run.

Protocol S3. Bash code for processing of ONT sequencing data. If all programs are readily installed, this code can be run without internet connection under field conditions. The code can be run on a portable local machine. However, availability of GPU resources for basecalling strongly enhances speed of the bioinformatic workflow. Any characters in *italics* correspond to variable file names or custom thresholds that need to be replaced with the desired names/parameters.

Basecalling with Dorado v0.9.0 in windows power shell using GPU mode (for CPU mode add `--device cpu`. Alternatively, basecalling can be done live using Dorado in MinKnow.

```
“\path\dorado\bin\dorado.exe” basecaller sup -r “\path\inputfolder” -o
“path\outputfolder” --emit-fastq
```

Now, switch to Linux environment

Deposit `minibar.py` script in your folder, make it executable and demultiplex with the following commands (make sure to deposit txt with index and primer data in the folder too). -T will trim off primer sequences, -F creates individual output folders. For details, check:
https://github.com/calacademy-research/minibar/tree/master

```
chmod 775 minibar.py
```

```
./minibar.py dmxLab.txt all_nanopore_files.fastq -F -T
```

We are creating a shell-script to filter all output files at once using Seqkit. -m specifies the min length, -M the max. length of the sequence to be written, -Q is the minimum quality score. Make sure to run separate filters on environmental (short) markers and specimen barcoding (long) markers.

```
ls *.fastq | awk '{print "seqkit seq -m 160 -M 180 -Q 12 "$0" >
"gensub(/\.fastq$/, "_filtered.fastq", "g", $0)}' > filter_fastq.sh
```

```
chmod +x filter_fastq.sh
```

```
./filter_fastq.sh
```

At this stage, we have to proceed differently for barcoding vs. metabarcoding data. We first have to process specimen barcoding data and supplement our BLAST database. We therefore create consensus sequences for specimen barcodes using NGSpeciesID. Make sure to manually sort the desired files in a new folder and change your directory accordingly. The following shell script will create individual output folders.

```
ls *_filtered.fastq | awk '{split($0, a, "_filtered.fastq"); print "mkdir -
p ./NGSpeciesID/" a[1] " && NGSpeciesID --ont --consensus --medaka --fastq
\"" $0 "\" --outfolder ./NGSpeciesID/" a[1]}' > run_NGSpeciesID.sh
```

```
chmod +x run_NGSpeciesID.sh
```

```
./run_NGSpeciesID.sh
```

Then, automatically extract your consensus sequences and name them after the sample folder using the `extract_fastas.sh` script.

Subsequently, merge them for a quick BLAST-check for taxonomic integrity using the following command:

```
for file in *.fasta; do [[ "$file" == "name.fasta" ]] && continue; awk -v
name="${file%.fasta}" '/^>/ {print ">" name; next} {print}' "$file"; done >
name.fasta
```

Although not mandatory, we recommend aligning and visually inspecting your consensus sequences at this stage to trim off erroneous sequence ends (NGSpeciesID is prone to create erroneous sequence overhangs beyond the actual length of your target marker). For alignment and curation, we recommend using mafft and CIAlign combined with a graphic program for visualization such as AliView or MEGA.

```
mafft –-adjustdirection name.fasta > name_aligned.fasta
```

```
CIAlign --infile name_aligned.fasta --outfile name_aligned_curated.fasta --
remove_insertions --remove_divergent
```

Now, simply paste your sequences in fasta format into your local GenBank (In our case: the MIDORI2 subset) data (if not downloaded in fasta format you can use the `blastdbcmd` command to transform it into fasta: `blastdbcmd -db 16S -entry all -outfmt "%f" -out 16S.fasta`). In practice, this means opening the fasta e.g. in a GUI editor and pasting the new sequences at the end or using the `cat` command to concatenate two fasta files. Then make sure to manually create the header of your sequence in a similar format so that BLASTn will later provide a meaningful taxonomic annotation (i.e. headers should look like: *NoID;Eukaryota_2759;Chordata_7711;Amphibia_8292;Anura_8342;Bufonidae_8382;Atelopus_47578;Atelopus_laetissimus_consensus*). Then, create a BLAST database from your supplemented fasta file:

```
makeblastdb -in name.fasta -dbtype nucl -out dbname
```

Check if BLAST can access the database.

```
blastdbcmd -db dbname -info
```

Now you can get back to your metabarcoding data, starting from the filtered fastq files. To reduce file size, change .fastq to .fas

```
ls *filtered.fastq | awk '{print "fastq_to_fasta -i \"" $0 "\" -o \""
substr($0, 1, length($0)-6) ".fas\" -n"}' > fas.sh
```

```
chmod +x fas.sh
```

```
./fas.sh
```

Now, dereplicate sequences

```
ls *filtered.fas | awk '{print "vsearch --derep_fulllength \"" $0 "\" --
output \"" substr($0, 1, length($0)-4) ".derep\" --sizeout --relabel Uniq"}'
> derep.sh
```

```
chmod +x derep.sh
```

```
./derep.sh
```

Instead of further clustering or denoising approaches, we recommend the direct-BLAST approach (Plewnia et al. 2025) for ONT data given that sequence error distance can exceed genetic distance between closely related species.

```
ls *.derep | awk '{print "blastn -db /path/dbname -query \"" $0 "\" -
max_target_seqs 1 -out \"" substr($0, 1, length($0)-4) "_OTUs.out\" -outfmt
```

```
\"6 qseqid sseqid stitle pident length mismatch gapopen qstart qend sstart send evalue bitscore staxids\""}' > blast.sh

chmod +x blast.sh

./blast.sh
```

The output can be read and processed with any GUI text editor and contains row-wise taxonomic annotations for each unique sequence. To generate an OTU table, weighted (by size of dereplication) sums can be built per taxon name using custom R code. An example can be found below in protocol S4.

Protocol S4. Example R script for downstream analysis of eDNA metabarcoding results, summarizing OTU/species read counts for all samples (based on the OTU tables with BLASTn results as generated and formatted in protocol S3). Characters in *italics* correspond to column names or custom thresholds that need to be replaced with the desired names/parameters. Note that, depending on the formatting of the taxonomy string, redundant symbols may persist in the taxon names, and a critical evaluation of the final results is required.

```
# Save and place script (.R file) in folder with all input OTU tables; set working directory to script location:
setwd(dirname(rstudioapi::getActiveDocumentContext()$path))

# Load Tidyverse (version 2.0.0 used for the script below):
library(tidyverse)

# List all OTU tables (named as .out files; see protocol S3) in the folder:
file_list <- list.files(pattern = "\\.out$")

### Process all OTU tables and return summarized read counts for each OTU/species, also retaining family designation. Numbers (#1, #2, #3) require specific column input (see more information below the script).
## Read the files (Here: as tab-delimited tables)
## Extracting & cleaning taxonomic data:
# — in this example: keep only records with a sequence similarity ≥ 95%
# — for sequence header containing "size=N"; extract N as numeric (read) count
# — extract family name from the taxonomy string (filtering for "dae" word ending)
# — extract species name (assuming they are the last semicolon-delimited field)
# — remove 'noise' extracted with the species and family names from the taxonomy string
# — replace the first underscore with a space ("Genus_species" → "Genus species")
# — remove remaining underscores, drop remaining digits
## Sum all read counts per OTU/species, with each column summarizing one input OTU table.

process_file <- function(file_name) {
 data <- read_tsv(file_name, col_names = FALSE)

 cleaned <- data %>%
  filter(column >= 95) %>% # (1)
  mutate(column = as.numeric(str_extract(column, "(?<=size=)\\d+")), # (2)
   family = str_extract(column, "\\w*dae"), # (3)
   family = str_remove(family, "^family_"),
   family = str_remove(family, "_\\d+$"),
   species = str_extract(column, "[^;]+$"), # (3)
   species = species %>%
    str_remove_all("(?i)^species_") %>%
    str_remove_all("_consensus") %>%
    str_remove_all("(?i)[sS][pP][0-9]*") %>%
    str_replace("_", " ") %>%
    str_remove_all("_") %>%
    str_remove_all("\\d+")) %>%

  select(family, species, count = column) # (2)

 summarized <- cleaned %>%
  group_by(family, species) %>%
  summarise(!!file_name := sum(count, na.rm = TRUE), .groups = "drop")

 return(summarized)
}
```

(1) Provide column containing sequence identity (% sequence similarity).
(2) Provide column containing the total read counts per OTU (specified as "size=N" after unique identification number as in our example).
(3) Provide column with full taxonomy string (formatted e.g. *NoID;Eukaryota_2759;Chordata_7711;Amphibia_8292;Anura_8342;Bufonidae_8382;Atelopus_47578;Atelopus_laetissimus_consensus*) as in our example.

Collate and clean all data:
— Replace NAs (species absent in some files) with 0 across columns
— Set all values ≤ 5 to 0 (regarding read counts below this threshold as sequencing noise)
— Remove taxa rows where the sum across all samples is 0 (after filtering)

```
all_data <- map(file_list, process_file) %>%
  reduce(full_join, by = c("family", "species")) %>%
  mutate(across(-c(family, species), ~replace_na(.x, 0))) %>%
  mutate(across(-c(family, species), ~ifelse(.x <= 5, 0, .x))) %>%
  filter(rowSums(across(-c(family, species))) > 0)
```

Save final summary table

```
write_tsv(all_data, "summary_table.txt")
```

Protocol S5. List of required equipment and considerations to complete the workflow under field conditions (adapted from Plewnia et al. 2025).

eDNA (water) sampling

- Portable water filtration pump (manual or battery-powered)
- Filter funnel compatible with filtration system (In this study: Nalgene, Thermo Scientific)
- Filters (In this study: 0.45 µm nitrocellulose filter, Sartorius; diameter as of filter funnel)
- Tweezers
- Gas or ethanol burner to sterilize tweezers
- Optional: sterilized bottles or plastic bags for water collection (samples can alternatively be taken directly in the water body)

Non-invasive amphibian sampling (to be adapted based on study system)

- Cotton-tip swabs

DNA extraction

While this paper was not aimed at improving field-based DNA extraction techniques, available methods involve various trade-offs around material needs, extraction time, DNA yields, and prices. Established extraction kits will yield higher quality DNA, but usually require more extensive equipment and time in the field and are more expensive. For instance, a heating device and centrifuge are required for the Qiagen DNeasy Blood & Tissue Kit and the Bio-Rad Chelex 100 Resin extraction protocol (see more considerations and trade-offs reviewed in Pomerantz et al. 2022; Ip et al. 2026). DNA extraction is alternatively possible using self-assembled gear and reagents. For example, following adaptations to the cellulose-based extraction protocol of Zou et al. (2018) as described in Plewnia et al. (2025), minimal requirements for field-based 'fast extraction' would be:

- A series of 1.5 ml screw-tubes with pre-prepared lysis, wash, and elution buffers (using Tris HCl, NaCL, EDTA, SDS, PVP-40, and silica-zirconium beads) and a binding tube with an 8 mm Whatman cellulose filter in a 3D-printed spin column.
- Sterile blades (for cutting up the filters)
- Portable oscillator/shaking device (for lysis using beads)
- Portable centrifuge

Recombinase Polymerase Amplification (RPA)

- RPA reagents (In this study: TwistAmp Basic kit, TwistDX; reagents can be pre-distributed into 0.2 ml reaction tubes before use in the field)
- RPA primers without index sequence (for amphibian metabarcoding, see manuscript)
- RPA primers with index sequence
- Optional: RNAse-free water (not needed if using maximum eluate volume)
- Optional: heater (small amounts of samples can be incubated at body temperature)

Nanopore MinION library preparation and sequencing

- Nanopore library preparation kit (SQK-LSK114)
- Portable centrifuge
- Adjustable heating device or thermal cycler
- Magnetic rack
- Magnetic beads
- Ethanol (80%)
- RNAse-free water
- 0.2 and 1.5 µl reaction tubes
- Qubit fluorometer (or equivalent) with reagents (to calibrate flow cell priming)
- MinION sequencer and flow-cells (e.g. Flongle flow cell with adapter)
- Laptop for MinION control and bioinformatic processing

General requirements and comments

- Sterile gloves to minimize contamination during all parts of the workflow and prevent pathogen transmission between sampled individuals.
- Sufficient 1.5 and 2 ml tubes and DNA preservative depending on planned sampling size, storage duration and further use of methods (In this study: DNA/RNA Shield, Zymo Research; for longer-term storage, not compatible with the cellulose-based extraction as described above).
- A series of adjustable (micro)pipettes (e.g. 0.5-10 µl, 2-20 µl, 10-100 µl, 100-1000 µl) and sufficient pipette tips to complete DNA extractions, RPA amplification, and Nanopore library preparation and sequencing given the number of samples. Pasteur pipettes and microcapillaries may alternatively be used to replace adjustable pipettes for most parts of the workflow (see Plewnia et al. 2025 and supplement for details).
- A portable external battery/power bank is highly recommended to avoid needing an electricity generator in the field to power equipment (e.g. Laptop, Qubit fluorometer, heating device, centrifuge) during relatively small-scale biodiversity assessments.
- Given the workflow listed above, the minimal ‘hardware’ to be taken into the field consists of:
    - eDNA sampling system
    - Oscillating/shaking device
    - Centrifuge
    - Adjustable heating device or thermal cycler
    - Qubit fluorometer (or equivalent)
    - Nanopore sequencer
    - Optional: a set of adjustable pipettes

Supplementary references

Chan, K. O., Hertwig, S. T., Neokleous, D. N., Flury, J. M., & Brown, R. M. (2022) Widely used, short 16S rRNA mitochondrial gene fragments yield poor and erratic results in phylogenetic estimation and species delimitation of amphibians. *BMC Ecology and Evolution*, 22, 37.

Higgins, M., Ravenhall M., Ward, D., Phelan, J., Ibrahim, A., Forrest, M. S., Clark, T. G., & Campino S. (2019). PrimedRPA: primer design for recombinase polymerase amplification assays. *Bioinformatics*, 35, 682-684.

Ip, Y.C.A., Allan, E.A., Hirsch, S.L., & Kelly, R.P. (2026) Fast, flexible, feasible: a transparent framework for evaluating eDNA workflow trade-offs in resource-limited settings. *Molecular Ecology Resources*, 26, e70091.

Lindenmayer, D., & Scheele, B. (2017). Do not publish. *Science*, 356, 800-801.

Palumbi, S. R., Martin, A., Romano, S., McMillan, W. O., Stice, L., & Grabowski, G. (2002). *The simple fool's guide to PCR* (Version 2.0). Kewalo Marine Laboratory, University of Hawaii at Manoa.

Plewnia, A., Krehenwinkel, H., & Heine, C. (2025) An isothermal workflow for low-cost and PCR-free field-based community metabarcoding. *Methods in Ecology and Evolution,* 16, 1413-1424.

Pomerantz A., Sahlin, K., Vasiljevic, N., Seah, A., Lim, M., Humble, E., … Prost, S. (2022) Rapid in situ identification of biological specimens via DNA amplicon sequencing using miniaturized laboratory equipment. *Nature Protocols*, 17, 1415–1443.

Portik, D. M., Streicher, J. W., & Wiens, J. J. (2023). Frog phylogeny: A time-calibrated, species-level tree based on hundreds of loci and 5,242 species. *Molecular Phylogenetics and Evolution*, 188, 107907.

Sakata, M. K., Kawata, M. U., Kurabayashi, A., Kurita, T., Nakamura, M., Shirako, T., … Minamoto, T. (2022). Development and evaluation of PCR primers for environmental DNA (eDNA) metabarcoding of Amphibia. *Metabarcoding and Metagenomics*, 6, e76534.

Zou Y, Mason MG, Wang Y, Wee E, Turni C, Blackall PJ, Trau M, & Botella JR (2018) Nucleic acid purification from plants, animals and microbes in under 30 seconds. *PLoS Biology*, 15, e2003916.
